# PROTOCOL: Causal mechanisms linking education with fertility, HIV, and child mortality: A systematic review

**DOI:** 10.1002/cl2.1250

**Published:** 2022-06-10

**Authors:** Fatima Zahra, Nicole Haberland, Stephanie Psaki

**Affiliations:** ^1^ Population Council Washington DC USA; ^2^ Population Council New York New York USA; ^3^ U.S. Department of Health and Human Services Washington DC USA

## Abstract

In this review, we will investigate the pathways linking education and health to understand why education appears to improve health in some settings or among certain populations, and not in others, as well as to inform recommendations about how best to target investments in education to maximize the benefits to health. We will seek to answer the following key research questions, focusing specifically on the mechanisms that affect fertility, HIV, and infant and child mortality. If feasible, these answers will include meta‐analyses of comparable education and mediator outcomes: (1) Do changes in education affect the primary theorized mediators (e.g., knowledge, attitudes, resources, and agency; health behaviors and harmful practices) of the relationship between education and fertility, HIV and child mortality? (2) How does the relationship between these mediators and education vary across different aspects of education (e.g., grade attainment vs. literacy/numeracy vs. attendance)?

## 1 BACKGROUND

### 1.1 The problem

In recent decades, governments and donors working in low‐ and middle‐income countries (LMICs) have made substantial investments in expanding access to school (UNESCO, 2015; World Bank, 2018). Beyond the goal of improving levels of attainment and learning, this investment has been based, in part, on an expectation that a better educated population would also be a healthier one. Yet despite enormous progress in expanding school enrollment, improvements in health have not always followed (Bongaarts & Casterline, 2013; B. Mensch et al., 2005). These patterns raise important questions: Does education, in fact, enable women, men and their families to be healthier? And if so, how?

A recent systematic review of the evidence for the effects of education on both sexual and reproductive health and maternal and child health in LMICs began to explore these questions (Psaki et al., 2019 on sexual and reproductive health; B. S. Mensch et al., 2019 on maternal and child health). Based on rigorous studies that estimated the causal relationships between education and health, the authors found that:

1. completing more years of school leads to women having fewer children;
2. more years of schooling has a protective effect against HIV for women; and
3. more years of schooling for women leads to a decrease in the risk of child mortality.

The authors also found that, despite assumptions about the effects of education on health, evidence is lacking on key aspects of this relationship. Specifically, they found limited or no evidence on the following relationships: the effects of education on sexual and reproductive health and child health outcomes for men; the effects of improvements in learning, including literacy and numeracy, on health for women or men; and the effects of education on maternal morbidity or mortality; among others. Furthermore, even in those analyses where sufficient evidence supported the effects of increased educational attainment on health outcomes, explanations for *how* this occurs were incomplete and often contradictory. These are important gaps in the evidence of the effects of education on health.

Many researchers, policymakers, and practitioners have speculated about how education may lead to improved health. To give an example of these hypothesized pathways, some argue that more years of schooling leads to stronger literacy, which enables women to navigate the healthcare system and provide better care for their children, resulting in lower child mortality (Glewwe, 1999). Others argue that the socializing experience of school, or the increased access to labor market opportunities offered by education due to skills or credentials, may change attitudes and preferences so that girls and women (and their families) prefer to delay marriage and childbearing and have fewer children (Duflo et al., 2015; Jensen, 2012). Those preferences, combined with the resources and skills needed to access family planning services, may lead to later marriage and childbearing, and lower fertility. Still others argue that agency (ability to act on one's own behalf) is a critical pathway linking education and health: those who complete more years of school may develop greater agency, leading to more equitable relationships, less intimate partner violence, and lower risk of HIV (Fielding‐Miller & Dunkle, 2017; Hanmer & Klugman, 2016; Yount et al., 2017).

Although the previous review mentioned earlier was not designed to test assumptions about the mechanisms linking education and health, many of the included studies explored these relationships (B. S. Mensch et al., 2019; Psaki et al., 2019). Yet results on these pathways were largely inconsistent. Two exceptions were: consistent, albeit correlational, evidence of a possible effect of literacy as a pathway linking grade attainment to health outcomes (Psaki et al., 2019), and reproductive behavior as a possible pathway linking women's educational attainment to child health (B. S. Mensch et al., 2019).

In short, despite theory, assumptions, and some empirical evidence, many of the purported pathways linking education and key health outcomes have not been systematically assessed and synthesized empirically. Understanding empirically and causally *how* education translates into improvements in health is essential not only to create better projections of likely improvements in health from recent gains in education, but also to understand the full impact and return of investments in education.

### 1.2 The  pathways

**Figure 1:**
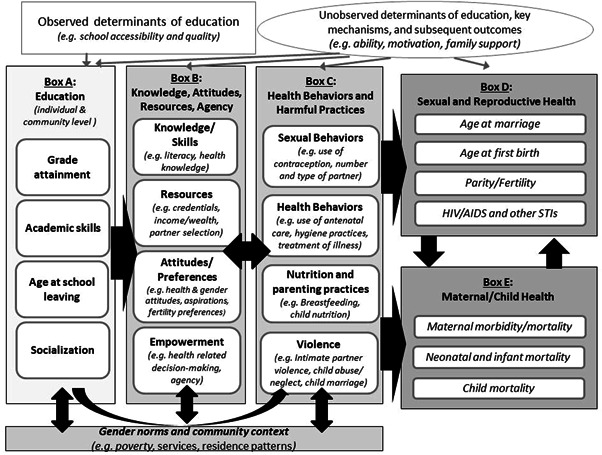
Conceptual framework of medium and long‐term pathways linking education to fertility, child mortality, and HIV status.

Figure 1 presents a conceptual framework, which lays out the hypothesized pathways linking education to medium and long‐term health outcomes including fertility, child mortality, and HIV status based on existing theoretical literature. We chose to focus on these outcomes given the findings from the recent systematic review that educational attainment leads to significant improvements in these three health outcomes (B. S. Mensch et al., 2019; Psaki et al., 2019).^1^ Evidence for a significant causal relationship makes understanding the pathways linking education to these outcomes even more compelling. We begin by providing an overview of the conceptual framework, followed by additional detail on the pathways linking education with the three health outcomes.

The Education box of the conceptual framework includes four components: grade attainment (the level of school completed), academic skills (often measured by literacy, numeracy or exam scores), age at school entry and leaving, and socialization (the effects of spending time in the school environment including with peers). Each of these are considered at the individual level and, in terms of general prevalence, at the community level. We include multiple indicators both because they may be linked to different causal pathways, and because they vary, often considerably, from one setting to another. For example, in settings with late entry to school, or high levels of grade repetition, the age of students may vary, even within the same level of attainment (Lam et al., 2013). Similarly, the skill levels of students vary within similar attainment levels, both across and within countries (World Bank, 2018). Some have argued that the experience of being in school—independent of the skills acquired or the grades attained, may also influence young people's attitudes and social networks in ways that affect health outcomes (Jejeebhoy, 1995).

The second box from the left also includes four domains: knowledge/skills (such as an understanding of how HIV is transmitted), resources (such as income, and partner characteristics/income), attitudes/preferences (such as fertility preferences, attitudes about gender roles in marriage), and empowerment (such as the ability to make decisions about a child's health, or ability to negotiate condom use). The third box, representing health behaviors and harmful practices/experiences, includes four components as well: sexual behaviors (including use of contraception), health behaviors (including seeking medical attention in case of illness), nutrition and parenting practices (including breastfeeding), and experience of violence (including intimate partner violence and harmful practices such as child marriage).

Underlying these first three boxes are gender norms and community context, including poverty, availability and quality of health services, disease prevalence, and norms related to education, gender roles, fertility, intimate partner violence, child marriage, and child health. These underlying factors may play a moderating role, determining the degree to which girl's better education outcomes can be utilized to achieve better health outcomes in a given setting. They may also interact directly with education—for example, if community norms support universal education, it is more likely that an individual girl will enroll in school, and similarly, the more individual girls stay in school, it may shift norms around girls' education. Norms and context may also interact directly with attitudes and agency—for example, if the norm is to have a child soon after marriage, individuals' attitudes around timing of first birth are more likely to reflect that norm. Finally, community norms and context interact directly with health behaviors and experiences. For example, in settings where it is normative to marry girls around age 16, it is more likely that a girl will be married as a child.

The health outcomes of interest are captured in two boxes, the first focuses on sexual and reproductive health, and includes age at marriage, age at first birth, parity/fertility, and HIV/AIDS and other sexually transmitted infections (STIs); and the second focuses on maternal and child health, including maternal morbidity/mortality, neonatal and infant mortality, and child mortality. Our framework presents the hypothesized pathways while acknowledging the layered, bidirectionality of some of these relationships.

In considering how researchers might estimate the role of each pathway, it is important to note several other factors (depicted at the top of the framework) that might influence these relationships: structural determinants of education, such as school accessibility and quality, which may or may not be measured in individual studies; and factors that may determine both education and health outcomes, many of which are often unobserved, including ability, motivation, and family support. Failure to account for unobserved factors that drive both education and health may lead to biased estimates of these relationships.

### 1.3 How the pathways might work

Education has been hypothesized to affect health outcomes through several direct and indirect pathways. Many of these mechanisms are shared for different health outcomes, although each outcome also has unique pathways. In addition, these pathways could interact in a variety of ways to impact health outcomes. For example, increased income alone may not impact health unless it is coupled with greater health knowledge. We primarily discuss pathways separately in this section, with more complicated interactions and synergies hypothesized in the final sub‐section.

#### 1.3.1 Knowledge and skills

Direct pathways from education to health include health knowledge acquired through formal education (Andrzejewski et al., 2009; Glewwe, 1999), and literacy and numeracy skills that assist in making healthcare decisions (Burchi, 2012; Glewwe, 1999; Smith‐Greenaway, 2013). Specifically, individuals may acquire knowledge related to multiple health domains while in school, including hygiene practices, pregnancy, contraception, the fertile period, child nutrition, the identification and treatment of diseases, and preventive measures like immunization (Ainsworth et al., 1996; Andalón et al., 2014; Argaw, 2013; Dinçer et al., 2014). School‐based programs that address sexuality, especially sex‐ and HIV‐education programs, can increase exposure to accurate information about contraception, reproduction, sexual health and HIV, and can lead to increased biological knowledge, including a more accurate understanding of reproduction, infection transmission routes, and prevention and treatment options.

In addition, literacy and numeracy skills gained through formal education may assist in acquiring new health information through greater capacity to understand instructions and messages from providers, community health workers, the news, or public health campaigns. Better comprehension of new health information may result in improved health behaviors, such as seeking and following the right medical treatment, engaging in safer sex, or adopting better child nutrition practices. A greater knowledge of science may lead to better ability to distinguish health facts from superstitions and rumors.

Education may also facilitate other skill‐based pathways. Social/soft skills such as learning to listen to a teacher, knowing how to follow instructions, or interacting with bureaucracies and people in positions of authority may enable one to have more successful interactions with health workers and the healthcare system later in life. Some pedagogical approaches are designed to foster critical thinking, an important academic skill that is useful in life, including in being more sophisticated consumers of health information, whether that mis/information is provided by providers, advertisements, or family members.

More educated women may thus be better equipped with the knowledge and skills to keep themselves and their children healthy, to follow instructions from medical professionals, and to interact with the healthcare system when needed to obtain care for ill children or sexual and reproductive health and HIV services for themselves (Basu, 2002).

In addition to these mechanisms that operate at the individual level, high community levels of education may affect health outcomes independent of individual levels of education. Specifically, less educated women may learn about hygiene practices and disease treatment that can reduce infant and child mortality from more educated mothers in communities where a greater proportion of women are educated (Kravdal, 2004; Vikram et al., 2012).

#### 1.3.2 Resources

Education can influence social and economic resources by leading to a change in socioeconomic status (Jukes et al., 2008). Women's and girls' control over resources, including their income, is also a potential pathway and is discussed in the empowerment section, below. Education provides better employment opportunities, and may encourage individuals to seek partners with similar levels of education (Currie, 2008; Keats, 2016). The former pathway is mostly straightforward: better employment may provide higher income that can be directed in part to the health and wellbeing of children and the family (Abuya et al., 2012; Currie, 2008; Kravdal, 2004). The latter mechanism is more complex. If more educated women and more educated men select (or are selected for) each other, they may jointly have greater earnings, but they may also be more aligned in their attitudes and preferences, as well as expectations regarding joint decision making (see below).

This latter resource pathway may thus have somewhat different manifestations depending on the health outcome. For example, for fertility, education may change the demand for children through partner selection if women marry more educated men who also desire fewer children, or who are more open to joint decision‐making about family planning (Basu, 2002; Du et al., 2016). Additionally, education may also lead to an increasing opportunity cost of childbearing for women who are able to earn an income in the labor market (Duflo et al., 2015; Güneş, 2016; Keats, 2016).

The resources that education can confer also have additional distinct hypothesized pathways to affect HIV risk. For example, selecting same age sexual partners instead of older, riskier, males has been shown to decrease unprotected sex (Dupas, 2011). Moreover, greater economic resources can decrease the likelihood of transactional sex (Austrian et al., 2019), which has been associated with HIV infection in Sub‐Saharan Africa (Wamoyi et al., 2016).

#### 1.3.3 Attitudes and preferences

Education may change attitudes, beliefs, and preferences, including attitudes toward using modern contraceptives, condoms for infection prevention, or modern medicine for maternal/child health or treatment of HIV (Basu & Stephenson, 2005; Glewwe, 1999). This may occur directly through school‐based life skills, health and/or sexuality and HIV education lessons, or empowerment or antiviolence programs, that aim to foster healthier behaviors over the life course by promoting positive attitudes (such as gender equitable attitudes or attitudes supportive of condom use and consent), providing accurate information, and countering falsehoods and mistrusts. The combination may help to ensure that individuals are both sufficiently motivated to avoid HIV infection or pregnancy and informed of different means of prevention (Fishbein, 2000; Jukes et al., 2008; Were, 2007). The influence may also flow via teachers and school administrators who often convey their own values, attitudes, and beliefs. In both these examples we note that such influence can operate in either direction. While some curricula and teachers have a progressive perspective that fosters belief in science and views contraceptives and condoms positively, other teachers and curricula are decidedly conservative, promoting abstinence only, misconceptions about vaccines, belief in fate, and so forth.

Education may also expose individual women and men to different family models that value fewer and more educated children (Caldwell, 1980; Grépin & Bharadwaj, 2015; Mocan & Cannonier, 2012; Verwimp, 2016), including by expanding access to mass media (Basu, 2002).

Expanded capacity—through resources, skills, and networks—to access television, advertisements, movies, newspapers, and social media, may expose people not just to smaller family models, but often also to relationships that are characterized by more open and equitable communication, and depict women (at least sometimes) in relatively less gender‐stereotyped roles (Gottert et al., 2020). Exposure to these alternative models may shift a person's attitudes, beliefs, and preferences, in positive or negative directions.

Similarly, education can also influence individuals' aspirations for the future, making them less inclined to engage in sexual activity that elevates their exposure to STIs and their risk of early childbearing (Clark et al., 2009). Multiple studies have demonstrated a link between educational and career aspirations and use of contraception, pregnancy, and childbearing. Qualitative work from Malawi also suggests that schooling can raise educational aspirations, often reframing girls' identity and prompting them to raise their marriage aspirations and avoid sexual activity and premarital pregnancy (Frye, 2012). Conversely, low levels of school attendance have been found to be associated with increased risk of incident herpes simplex virus 2 (HSV‐2) infection due to decreased aspirations for the future and age‐disparate sexual partnerships (Stoner et al., 2019).

#### 1.3.4 Empowerment

Empowerment is both a process of transforming power relations and an outcome. It has been defined as the expansion of people's ability to make strategic life choices where such choices were previously denied, to increase their voice in discussions both public and private, and to act on their own behalf to achieve desired outcomes (Kabeer, 1999; van Eerdewijk et al., 2017). For clarity, we divide related concepts and measures into the power to achieve choices, the power over resources, power within the individual, and power with others.

Education may foster, particularly among women, greater power to achieve choices along with greater power over resources that enable those choices. This may occur through an improvement in their bargaining power within households and relationships and enabling them to make health‐related decisions for themselves and their children (Behrman, 2015; Caldwell, 1986; Fantahun et al., 2007) and to negotiate safer sex, contraceptive use, and smaller family sizes with partners (Jewkes et al., 2003; Keats, 2016; Mocan & Cannonier, 2012; Wolff et al., 2000).

Crucially, education may inculcate power within through agency and self‐efficacy. Agency—“the ability to define one's goals and act upon them” (Kabeer, 1999)—is central to most definitions. More educated people are more likely to believe they have control over their behavior, rather than another individual or fate, and thus are more likely to exert control over their own behavior (Maercker et al., 2019). Women with more education may also have more autonomy and voice in their communities, enabling them to interact effectively with the healthcare system and providers, and gain access to contraception and other health commodities and services (Jejeebhoy, 1995; LeVine et al., 2012; Vikram et al., 2012).

We note that empowerment and agency (as well as gender attitudes and gender norms), are sometimes conflated and inconsistently measured, often operationalized by narrow indicators rather than broader measures that might better capture the full construct. Other measurement challenges in this domain include relevant indicators being excluded where necessary. This becomes challenging if, for example, studies use household decision making as their measure of agency and find no impact on health behaviors such as condom use, whereas if they instead had operationalized agency as the ability to insist on condom use, they might have.

Finally, high community levels of education offer an additional pathway whereby less educated women are exposed to educated mothers who may model greater agency in health‐related decision‐making, and advocate for better healthcare in their communities. Education may thus foster power with other women to enable change at institutional, community, and household levels.

#### 1.3.5 Health behaviors and harmful practices

While we hypothesize that Box A is linked to Box C primarily through Box B, education may also be linked to health behaviors and harmful practices directly. At a basic level, school attendance, regardless of learning outcomes, may impact students' health behavior through the structure it imposes on students' time use patterns (Jukes et al., 2008). This pathway thus skips the intermediate knowledge, attitude, resources, and empowerment domains and links education directly to health behaviors and harmful practices. Thus, viewed over the course of adolescence, education is hypothesized to serve as a mechanism that simply delays transitions to adult roles, such as sexual debut, childbearing, and marriage, (Bongaarts et al., 2017; Santelli et al., 2015) often referred to as the “incarceration effect” of schooling (Black et al., 2008; Tequame & Tirivayi, 2015; Verwimp, 2016).

The experience of simply being in school also includes friendship and peer networks. Social networks are considered especially important for adolescents and young people because of the power of peer norms to influence behavior (Albarracín et al., 2004). However, while it has been hypothesized that education may act as a protective mechanism by sorting people into safer sexual networks, a reverse scenario—of those networks promoting riskier behaviors—is indeed possible (Jukes et al., 2008; Lam et al., 2013). Similarly, school attendance may result in direct exposure to school‐based violence perpetrated either by peers or teachers.

#### 1.3.6 Norms, household, and community context

Contextual factors such as poverty and social an gender norms underlie or intersect with many of the above pathways at the household and community levels. Specifically, both gender and social norms at the community level (Maertens, 2013), and parents' own attitudes and preferences may influence parents' decisions about girls' schooling, child marriage, the acceptability of premarital sex, as well as girls' aspirations and work opportunities, which may contribute to the pathways from education to health outcomes at multiple points (Mathur et al., 2001).

At the community level, norms around girls' education and the value placed on girls will determine whether a girl stays in school or attends school in the first place. Gender and social norms in the community can influence individual attitudes and aspirations, as well as the likelihood of a woman being able to translate her higher education into employment opportunities. Norms around marriage may contribute to premature school dropout in addition to the timing of marriage and choice of partner, as may norms regarding the compatibility of schooling and premarital pregnancy (Frye, 2017; Grant & Hallman, 2008).

Other contextual factors, such as poverty or the proximity of affordable, quality health services, may also increase or decrease the likelihood that better education outcomes can be utilized for better health outcomes. Similarly, at the school level, in settings where school‐based health programs are available, attending school can facilitate students' access to health services, including HIV prevention and testing, either directly (through school‐based clinics) or through referrals.

At the household level, poverty may influence both educational outcomes and Box B and C (see Figure 1). For instance, poor households may curtail education in favor of child marriage, which then increases the risk of poor maternal and child health (Chari et al., 2017). Similarly, household poverty may increase the likelihood of engaging in risky, transactional sex, particularly among girls who drop out of school. As another example, gender bias and preferences at the household level may result in gender differences in children's educational aspirations (Favara, 2017), which then affect sexual behavior (Frye, 2012). Other aspects of the household, including household formation patterns may interfere or accelerate the leveraging of education for health. For example, where young couples reside in extended family households, the influence of family members in determining whether a young woman receives antenatal care, provides colostrum to her newborn, or takes a job, will be substantially higher than in settings where young couples reside in nuclear families. Conversely, the childcare support in extended families may make taking a job, or bringing a younger child to health services, more feasible.

#### 1.3.7 Complex interactions

For several of the relationships depicted in this framework there are additional interactions that amplify or reinforce effects. For example, education may influence fertility as outlined above, but may also do so indirectly via reduced infant and child mortality rates because women and their partners need to have fewer births to end up with their desired number of living children (Cleland, 2010; Gakidou et al., 2010; Shapiro & Tenikue, 2017). Conversely, the framework also shows that fertility decisions affect infant mortality, mainly through age at birth, birth spacing practices, and birth order (Frost et al., 2005; Hobcraft, 1993; Madise et al., 2003). In addition, low birth weight, which is associated with fertility and health behaviors, is also associated with child health and mortality (Currie, 2008).

Hypothesized mediators may interact in complicated ways. For example, increased agency may not reduce early marriage (and hence fertility) unless the young woman also holds more equitable gender attitudes (Kenny et al., 2019). There are also ripple effects on the pathway from lower educational attainment to child marriage. For child marriage, in turn, is not only associated with earlier and more frequent childbearing, but also with less equitable relationships, and elevated risk of experiencing intimate partner violence (IPV) (Malhotra & Elnakib, 2021). Other examples include IPV, which is not only associated with a greater likelihood of infant and child mortality (Silverman et al., 2011) and HIV risk (Dunkle et al., 2004; Li et al., 2014), but may reinforce inequitable gender attitudes and women's low power in relationships.

### 1.4 Justification for review

#### 1.4.1 Informing policy and practice

Sustainable Development Goal (SDG) 3 focuses on ensuring the health and well‐being of people of all ages and is thus recognized as an important part of the global development agenda. This goal aims to push the global community to ensure universal access to family planning, eliminate the HIV/AIDS epidemic, and reduce infant and child mortality to 12 and 25 deaths per 1000 live births, respectively. However, despite important reductions in infant and child mortality, progress toward achieving family planning, adolescent birth, and HIV targets has been slow (UN General Assembly, 2015). Given that most of these outcomes disproportionately affect girls, their progress is also tied to the achievement of SDG 5, which focuses on achieving gender equality and empowering women and girls.

Recognizing the potential for education to improve health outcomes, the “UNESCO Strategy on Education for Health and Well‐being” (2016) has outlined policies and programs aimed at strengthening this link. For instance, the strategy emphasizes the continued need for policies promoting comprehensive sexuality education (CSE) including HIV education, and highlights the importance of including education on nutrition and physical health in school curricula (UNESCO, 2016). However, there is wide variation in the quality and implementation of health curricula, with those who drop out of school early often missing important health information taught at later grades. Additionally, while such policies acknowledge the direct pathways that link education to some health outcomes, it is unclear, given recent evidence, whether they are ultimately effective in improving health outcomes later in life.

While recommendations will vary based on what we find, unpacking pathways of change will allow us to identify interventions that may offer multiple benefits and are thus key investments for helping countries reach their SDG targets, as well as to identify those factors that, despite compelling theory, do not catalyze as diverse change. We hope this will allow us to provide recommendations for how education investments should be targeted as well as for program design. We will also identify potential important pathways that lack empirical support to inform future research directions and recommend methodological(including measurement) adaptations that may allow future studies to better capture mediation effects.

#### 1.4.2 Prior reviews

Previous reviews, some systematic, have focused on both causal evidence and associations between education and health outcomes in low and middle‐income countries. As noted previously, one recent systematic review highlighted important gaps in causal evidence on the relationship between education and health (B. S. Mensch et al., 2019; Psaki et al., 2019). The authors found that higher educational attainment leads to having fewer children, lower risk of HIV for women, and lower child mortality. However, the review also found that effect sizes tended to be small, which contradicts policy efforts to invest in education with the expectation of making substantial improvements in health outcomes. To our knowledge, no previous systematic reviews have evaluated the mediators of the relationship between education and fertility, HIV, and child mortality.

In addition, previous reviews have focused mainly on educational attainment, or specific types of education, such as school‐based sex education programs or compulsory schooling laws (Fonner et al., 2014; Hamad et al., 2018). Few have reviewed studies that examine other aspects of education, including literacy, numeracy, attendance, and age at school leaving (Psaki et al., 2019). Other reviews focused on LMICs have found that higher female education is associated with lower fertility in South Asia, noted a shift in the association between education and HIV prevalence, and found that participation in sex education programs reduces HIV risk (Fonner et al., 2014; Hargreaves & Glynn, 2002; Sheikh & Loney, 2018). However, these reviews mainly examine *associations* instead of causal evidence, focus on specific programs or regions, and provide little evidence on the causal mechanisms that mediate the relationship between education and health outcomes in all LMICs.

#### 1.4.3 Contribution of this review

This review departs from those that already exist in that we aim to understand the causal pathways linking education to three health outcomes, which have been shown to be causally linked with educational attainment: fertility, HIV status, and child mortality. We will include studies that were captured in the previous review by Psaki et al. and Mensch et al. that did look at mediation effects, newer mediation studies that were not available at the time of the earlier reviews, as well as other studies that establish a causal link between education indicators and hypothesized mediators and are conceptually linked to health outcomes even if the study does not quantitatively follow the full path to a health outcome. Studies that analyze these relationships may take the form of randomized controlled trials (RCTs) or quasi‐experimental analyses of programs and policies, as detailed below.

As the global community pushes for better integration between SDGs, and as policymakers seek to address the linkages between development challenges faced by their populations, the need to identify the most effective interventions is more pressing than ever. This review is designed to fill a gap in existing evidence on when and how education may translate into improved health outcomes to inform more effective investments.

Through this review we will identify hypothesized pathways linking education to these three health outcomes for which:

- The evidence is strong (either in support or against)
- The evidence is weak or non‐existent; and
- Gaps in the evidence exist but the links are promising, such that further investment in understanding those connections is warranted.

The contribution of this systematic review is to build on our understanding of the causal links between education and health outcomes to better understand how and why education is most likely to lead to improvements in health.

## 2 OBJECTIVES

In this review, we will investigate the pathways linking education and health to understand why education appears to improve health in some settings or among certain populations, and not in others, as well as to inform recommendations about how best to target investments in education to maximize the benefits to health. We will seek to answer the following key research questions, focusing specifically on the mechanisms that affect fertility, HIV, and infant and child mortality. If feasible, these answers will include meta‐analyses of comparable education and mediator outcomes:

1. Do changes in education affect the primary theorized mediators (e.g., knowledge, attitudes, resources, and agency; health behaviors and harmful practices) of the relationship between education and fertility, HIV and child mortality?
2. How does the relationship between these mediators and education vary across different aspects of education (e.g., grade attainment vs. literacy/numeracy vs. attendance)?

## 3 METHODOLOGY

**Figure 2:**
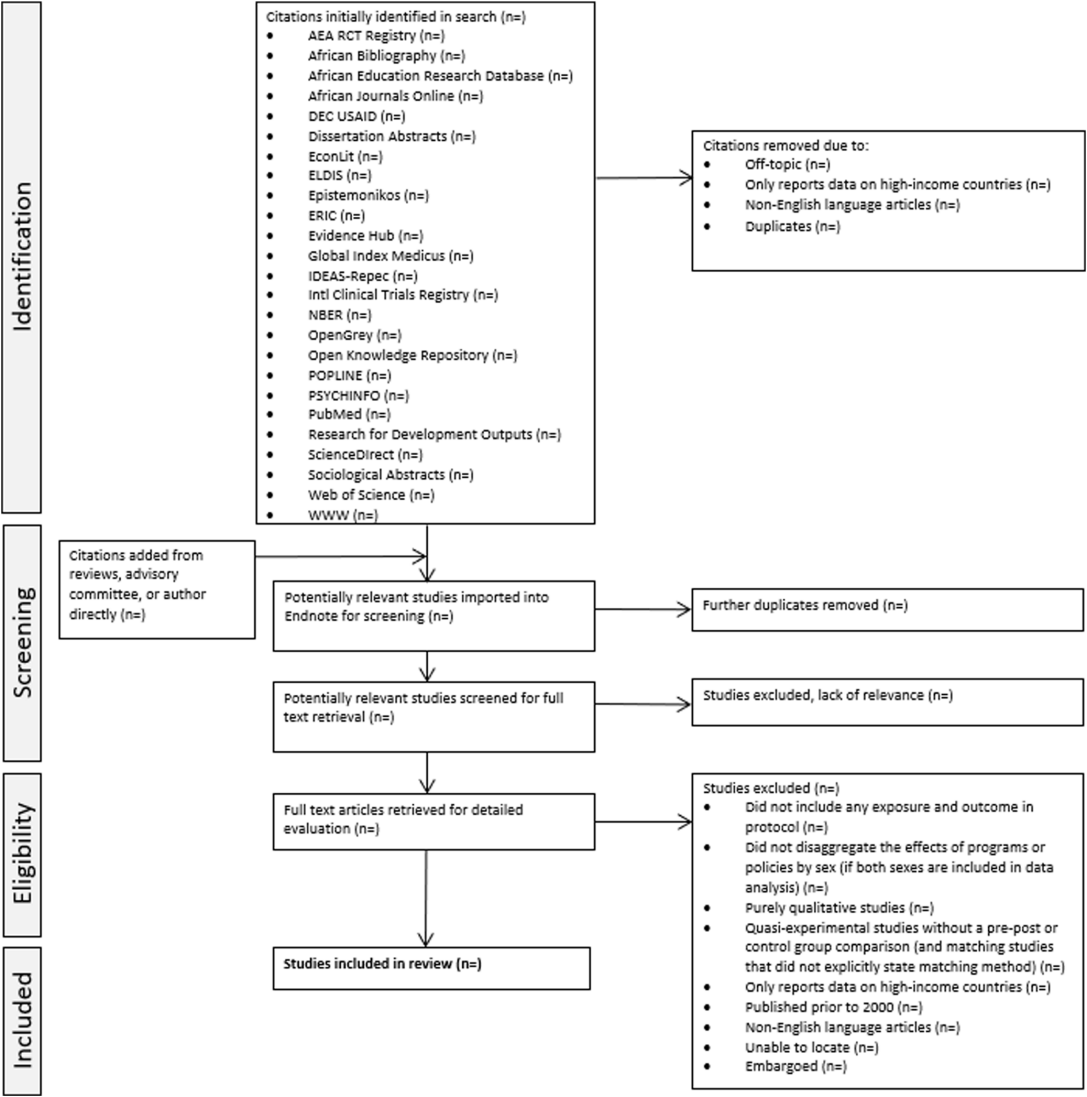
PRISMA flow diagram

The following section outlines our methodological approach to the review. These methods are largely based on the Cochrane Handbook, *Practical Meta‐Analysis* by Mark Lipsey and David B. Wilson, and *Research Synthesis and Meta‐Analysis: A Step‐by‐Step Approach* by Harris Cooper (Cooper, 2015; Higgins & Green, 2011; Lipsey & Wilson, 2001) (Figure 2).

### 3.1 Criteria for including and excluding studies

To assess the current evidence on causal links between education and fertility, HIV status, and child mortality, studies published before 1990 will be excluded. We will only include studies in English.

#### 3.1.1 Types of study designs

Studies that attempt to control for endogeneity^2^ through one or more of the following methods will be eligible for inclusion^3^:

- RCTs (longitudinal data, or post‐intervention data for studies with large sample sizes^4^)
- Regression discontinuity (longitudinal or cross‐sectional data)
- Instrumental variables analysis (longitudinal or cross‐sectional data)
- Difference‐in‐differences analysis (longitudinal or cross‐sectional data)
- Other quasi‐experimental studies with either:
  o a matched comparison group where the matching procedure is described (e.g., nearest‐neighbor matching, propensity score matching) OR
  o a comparison group where quasi‐treatment and quasi‐control groups are either stratified or tested for balance, both based on more than one sociodemographic characteristic that are justified by background literature
  o Synthetic control analysis
- Interrupted time series (longitudinal data)

While RCTs are largely considered the gold standard when evaluating the effect(s) of a given intervention or exposure, exogenous changes in policies and exposures that act as quasi‐experiments can also provide valuable insight when evaluating said effect(s), particularly when paired with quantitative methods that attempt to control for endogeneity. Thus, studies reporting on both RCTs and quasi‐experiments are eligible for inclusion. Articles that utilize study designs outside of those listed above will be excluded, notably:

- RCTs that only report post‐intervention data from a small‐scale intervention
- Quasi‐experimental studies without a pre–post or quasi‐control group comparison.
- For studies that employ matching, no formal matching method was stated.

We will evaluate interventions or exposures with program and policy effects on all key study outcomes using the following criteria:

- The same exposures and outcome measures must be analyzed for both treatment and control groups (experimental or otherwise);
- Evaluations must include and report on all of the following:
  o A comparison or control group (either experimental or quasi‐experimental),
  o Randomization of program exposure or proper analytical approach(es) to address the selectivity of program participation (to best determine causality).
  o Conducted using data from LMICs based on World Bank definitions;
  o Report on the results of exposures or interventions targeting primary, secondary, and/or tertiary school‐aged youth;
  o Published in 1990 or later.

#### 3.1.2 Types of participants

The primary focus of this review will be females and males (and their children) in LMICs, as defined by the World Bank.

#### 3.1.3 Types of interventions/exposures

Studies must analyze interventions or exposures included in Box A of the conceptual framework to be included:

1. School enrollment or attainment, including:
  - Grade attainment (e.g., continuous, where every unit increase is equal to an additional grade the individual progressed to).
  - Years of schooling (e.g., continuous, where every unit increase is equal to an additional year of schooling)
  - Enrollment in primary school (e.g., dichotomous, =1 if enrolled, =0 if otherwise)
  - Enrollment in secondary school (e.g., dichotomous, =1 if enrolled, =0 if otherwise)
  - Enrollment in university/tertiary education (e.g., dichotomous, =1 if enrolled, =0 if otherwise)
2. Learning, including:
  - Academic skills (literacy and numeracy, exam scores) during and after leaving school (e.g., dichotomous, =1 if able to read a paragraph prompted by the data collector, =0 if otherwise)
  - Critical thinking skills (e.g., Test of Science Critical Thinking [TSCT]; Mapeala & Siew, 2015)
3. Age at school leaving, measured as continuous age or dichotomous (e.g., dropped out before age X)
4. Socialization in school, including: the influence of peer norms (e.g., measured as attitudes/perceptions of norms, or aggregate attitudes at the school community level)
5. Interventions that target barriers to schooling and impact the measures stated above.

#### 3.1.4 Types of outcome measures

Studies that report results for the primary outcomes listed below will be included. Studies that do not include any of the primary outcomes below in their analyses will be excluded. The inclusion of the primary outcomes will also be conditional on Box D and E. In other words, primary outcomes must be linked to fertility, child mortality, and HIV either with those health outcomes explicitly measured in the same study, or indirectly ‐ for instance, attitudes about HIV, or decision‐making regarding contraceptive use or child health. Outcomes of interest are to be measured in all comparison groups whether pre/post or intervention/control.

##### Primary outcomes

Since this review examines pathways, we are particularly interested in the potential mediating variables (such as attitudes, empowerment, etc.). Thus the potential mediating variables—rather than ultimate outcomes of fertility, HIV, and child mortality—are elaborated here as our primary outcomes. The bullets are examples in each category, but do not represent a comprehensive list. For example, any measure of knowledge and/or skills would be included, not just those listed below. Measures listed for each outcome below are how they are commonly assessed. Studies will not be excluded if they do not measure the outcomes in the way we outline below.

*Knowledge and skills*:

- Fertility, HIV, or child health‐related knowledge (e.g., continuous score on a knowledge assessment such as correctly stating how HIV is transmitted)
- Literacy, numeracy, or other academic skills (e.g., score on assessment administered by interviewers, self‐reported literacy or numeracy, performance on school exams)

*Resources*:

- Academic or other credentials (e.g., dichotomous completion of primary school, secondary school diploma)
- Financial resources (e.g., household ownership of assets, monthly income, savings)
- Partner selection (e.g., partner's education level, partner's monthly income)

*Attitudes and preferences*:

- Health attitudes (e.g., responses on a scale measuring attitudes toward care‐seeking when ill for oneself or one's child, whether it is acceptable to use contraception)
- Preferences, including fertility (e.g., preferred number of children, continuous)
- Gender attitudes (e.g., responses on a scale measuring gender attitudes, including attitudes toward violence against women)
- Aspirations (e.g., future aspirations, career aspirations)

*Empowerment*:

- Power to and power over: Health‐related decision‐making (e.g., who decides to seek care for oneself or one's child when ill, relationship power)
- Power within: Agency (e.g., control over financial resources, mobility, condom use agency, self‐efficacy)
- Power with: Participation or leadership in relevant local groups

*Sexual behaviors*:

- Contraceptive use (e.g., dichotomous measure of having ever used modern contraception or current use of modern contraception)
- Infection prevention (e.g., dichotomous measure of having used a condom at last sex)
- Number and type of partner (e.g., continuous measure of sexual partners in the previous 12 months, dichotomous measure of casual partner in the last 12 months (Y/N))
- Transactional sex (e.g., dichotomous measure of ever having sex in exchange for gifts or money)

*Health behaviors*:

- Use of antenatal and other maternal care (e.g., number of antenatal care visits during pregnancy, dichotomous measure of whether there was a skilled attendant at birth)
- Hygiene and sanitation practices (e.g., availability of clean water and soap in the home, frequency of handwashing)
- Treatment of illness (e.g., taking child with persistent fever to a healthcare professional)
- Immunization (e.g., child is up to date on all recommended immunizations)
*Nutrition and parenting practices*:
- Breastfeeding (e.g., exclusive use of breastmilk to feed child after birth, months of exclusive breastfeeding)
- Child nutrition and stunting (e.g., height for age *z*‐score, weight for height z‐score; stunting, wasting)
- Parenting (e.g., time spent reading to child, time spent playing with child)
*Violence*:
- Intimate partner violence (e.g., dichotomous measure of having ever experienced physical or sexual violence from an intimate partner in the last 12 months)
- Child abuse/neglect (e.g., dichotomous measure of having experienced physical or sexual violence as a child)
- Child marriage (e.g., continuous measure of age at marriage, dichotomous measure of having married before age 18)

##### Secondary outcomes

None.

#### 3.1.5 Duration of follow‐up

The authors will not exclude studies based on duration of follow‐up but will note duration in the data extraction table.

#### 3.1.6 Types of settings

Studies that report on the primary outcomes listed above using data from LMICs at the time of the intervention/exposure, as defined by the World Bank, will be included. Studies that only report on outcomes from high‐income countries, as defined by the World Bank, will be excluded.

### 3.2 Search strategy

The following databases will be searched electronically:

**Table:** 

Database name	Platform	Web address
AEA RCT Registry	AEA	https://www.socialscienceregistry.org/trials/search?
Africa Bibliography	Cambridge UnivPress	https://africabibliography.cambridge.org/
Africa‐Wide Information	EBSCO	https://www.ebsco.com/products/research-databases/africa-wide-information
African Education Research Database	REAL Centre, ESSA	https://essa-africa.org/node/501?action=searchadvanced
African Journals Online	AJOL	https://www.ajol.info/index.php/index/search
CAB Global Health	Ovid	https://www.cabi.org/publishing-products/global-health/
DEC USAID	USAID	https://dec.usaid.gov/dec/content/AdvancedSearch.aspx?
Dissertation and Theses Global	ProQuest	https://search.proquest.com/...genre=dissertations+%26+theses%26sid=ProQ:ProQuest+Dissertations+%26+Theses+Global
EconLit	EBSCO	https://www.ebsco.com/products/research-databases/econlit
ELDIS	IDS	https://www.eldis.org/search
Epistemonikos	Epistemonikos	https://www.epistemonikos.org/en/advanced_search
ERIC	EBSCO	https://search.ebscohost.com/login.aspx?direct=true%26db=eric
3ie Development Evidence Portal	3ie	https://developmentevidence.3ieimpact.org/
Global Index Medicus	WHO Global Health Library	http://www.globalhealthlibrary.net
Intl Clinical Trials Registry	WHO ICTRP	https://trialsearch.who.int/AdvSearch.aspx
ISRCTN Registry	ISRCTN	https://www.isrctn.com
Medline	EBSCO	https://www.nlm.nih.gov/medline/medline_overview.html
NBER	NBER	https://www.nber.org/papers.html
PsycINFO	EBSCO	https://search.ebscohost.com/login.aspx?direct=true%26db=psyc
RePEc	EBSCO	http://repec.org/ (NOTE: though this resource is available via this link, we searched via EBSCO)
Research for Development Outputs	FCDO R4D	https://www.gov.uk/research-for-development-outputs
ScienceDirect	Elsevier	https://www.sciencedirect.com/search?
Sociological Abstracts	ProQuest	https://search.proquest.com/…genre=article%26sid=ProQ:ProQ%3Asocabs
Web of Science	Clarivate	https://clarivate.com/webofsciencegroup/solutions/web-of-science/
World Bank eLibrary	EBSCO	https://elibrary.worldbank.org/search/advanced (NOTE: though this resource is available via this link, we searched via EBSCO)

Gray literature will be identified using the databases listed above, that is, African Education Research Database, DEC, ELDIS, NBER, and R4D. Only studies published in English will be included. Additional unpublished/ongoing studies will be identified through searches of websites of specific organizations identified in the search, to be key resources. These organization websites will include: Center for Global Development, CARE, CEDPA, High‐Quality Technical Assistance for Results (HEART), International Center for Research on Women (ICRW), J‐PAL (Poverty Action Lab), APHRC, Population Council, FHI 360, RTI International, UNESCO, UNGEI, UNFPA, and UNICEF. References from these websites will be reported in a general category (World Wide Web, i.e., WWW). Additional organizations we identify through the search may be added to this list.

The search strategy documented in Supporting Information: Appendix Section A will be used to conduct searches through ERIC and will be adapted to conform to the search functions of the other databases. Reference lists and bibliographies in relevant review articles and reports of systematic reviews found in the search will also be combed to identify additional articles eligible for inclusion. Reviewers will also contact relevant researchers and organizations identified through the search as key resources in the field to locate additional articles eligible for inclusion.

### 3.3 Details of study coding categories

#### 3.3.1 Article screening

Articles will be identified through searching the databases listed in the search strategy shown in Supporting Information: Appendix Section A. Initial screening will remove duplicate entries. After duplicates are removed, each title and abstract will be screened by two reviewers based on the inclusion criteria documented above through Covidence. In the case of any disagreements, the two reviewers will meet to discuss the article. If they do not resolve their disagreement, a third team reviewer will settle disagreements regarding study inclusion.

Before the full text review, a sample of ten randomly selected articles will be jointly reviewed by the project team and any disagreements will be discussed to establish that interpretation of the content of each article is similar for the two reviewers. Randomized selection of articles will be performed in Stata 16. Once the final set of abstracts is agreed upon, full text will be linked to each article. Full text will be reviewed by two reviewers, following the same screening and disagreements resolution procedures as the title and abstract review.

#### 3.3.2 Data extraction

Data extraction, along with intervention grouping, will be completed by two reviewers through an online tool (Google Forms); instructions for the form can be found in Supporting Information: Appendix Sections D and E. The data extraction form has been designed in consultation with the Cochrane Handbook (Higgins & Green, 2011) and adapted from Psaki et al., and B. S. Mensch et al. (2019). Where data are missing that could determine the inclusion eligibility of a study, such as effect size conversion, reviewers will contact the study author(s) to request the relevant information; three attempts to contact the author(s) will be made within 1 month. If the author(s) does not respond or does not provide the relevant information within 1 month of the first date of contact, then the study will be excluded from quantitative synthesis but may be included in narrative synthesis. Tables 1 and 2 provide an example subset of the data extraction form meant to provide ease of readership and may change depending on journal requirements. The complete data extraction form will be included as a part of supplementary materials on Dataverse.

**Table 1:** Characteristics of included studies

Title	Authors	Type of publication	Countr(ies)	Dates of data collection	Study design	Analysis method(s)	Study population	Age range	Sample size	Gender disaggregated results included
										

**Table 2:** Results—Effects

Study	Country	Age range	Sample size	Attrition/Response rate (if RCT)	Intervention/Exposure	Intervention/exposure measure (dichotomous vs. continuous)	Frequency and duration of exposure	Single versus multi‐component	Components included	Education outcome	Outcome measure (dichotomous vs. continuous)	Standardized effect size (95% CI)
												

#### 3.3.3 Risk of bias assessment

We will perform an assessment of risk of bias in both experimental and quasi‐experimental studies adapted from RoB 2 (Higgins et al., 2016) for randomized studies and ROBINS‐I (Sterne et al., 2016) for non‐randomized studies. Risk of bias will be assessed by two reviewers. Any disagreements will be discussed and if the initial two reviewers cannot agree, a third reviewer will assist in resolving any disputes. As a part of the non‐randomized studies tool, we include methods‐specific criteria from Psaki et al. (2019), which were adapted from Baird et al. (2013). While RCTs are generally considered the gold standard for identifying causal impact, they may still be subject to threats to validity (e.g., differential loss to follow‐up and selective reporting of outcomes). Given the larger body of literature that utilizes quasi‐experimental methods, our risk of bias assessment tool will incorporate criteria for both experimental and quasi‐experimental studies. The full assessment of risk of bias tool is presented in Appendix Section B. The relevant section of the risk of bias assessment tool will be applied to each included study at the time of data extraction.

### 3.4 Quantitative analyses

We will first provide a narrative summary of characteristics of included studies, including country, type of publication, sample size, the type of analysis conducted, estimation procedures used, and our assessment of risk of bias. We will then provide quantitative summaries of the findings grouped by exposure and outcomes, converting each result into a partial correlation. Details on effect size conversion, method of analysis, assessment of publication and small study bias, as well as sensitivity analyses procedures are provided below.

#### 3.4.1 Statistical procedures and conventions

We expect that the vast majority of effect sizes presented in the included set of studies will be in the form of unstandardized regression coefficients. All effect sizes will be converted into partial correlations for the purposes of this quantitative analysis. Due to the anticipated diversity of covariates used in the models within our expected pool of included studies, we choose to convert to partial correlations as it represents the relationship between two variables, controlling for (i.e., “partialling out”) covariates, and range in value from −1 to 1 (Aloe, 2014; Aloe & Thompson, 2013). Corresponding 95% confidence interval (CI) will be calculated for all outcomes if not already provided, and estimates will be presented in either tabular form or a forest plot.

We include equations to convert effect sizes, standard errors, and 95% CI in Supporting Information: Appendix Section C, which are drawn from Aloe and Thompson (2013), The Campbell Collaboration (Polanin & Snilstveit, 2016), and formulas developed in consultation with David B. Wilson for the Psaki et al., and B. S. Mensch et al. (2019) reviews.^5^ Conversion equations will be chosen based on the available information in each included paper.^6^ To maintain comparability of results, we will use one conversion equation for all effect sizes grouped by the criteria below where possible,^7^ defaulting to the equation where all studies within each group and model type provide the same information necessary for conversion.

Effect sizes of studies will be grouped and analyzed based on the following study characteristics:

- Intervention/Exposure (grouping criteria taken from the *Types of Interventions/Exposures* section):
  o The level of implementation of the intervention is the same (individual, household, school, hospital/clinic, other community‐level, other).
- Randomized versus Quasi‐experimental study
- Education Outcome
- Unit of analysis

We will not quantitatively combine the results of randomized and quasi‐experimental studies into the same meta‐analyses, nor will we combine interventions that fall into different categories in the “Types of interventions/exposures” list.

In addition, some studies may be subject to unit of analysis error, where the unit of analysis does not match the unit of allocation (Higgins & Green, 2011). We follow the Cochrane Handbook recommendations for studies that do not correct for one or more of these types of errors, but a summary of how we will treat the results subject to various manifestations of unit of analysis errors is provided below.

- Cluster randomized trials:
  o For cluster randomized trials that do not adjust for clustering in their results, given number of clusters, outcome data, and an estimate of ICC, we will adjust the analyses by dividing the reported sample size by the design effect,
1+(M−1)ICC1+(M−1)ICC,
  where *M* is the average cluster size and ICC is the intraclass correlation coefficient. If average cluster size and/or intraclass correlation is not provided in the paper, we will exclude the results of that paper (Rao & Scott, 1992).
- Cross‐over trials
  o While results from cross‐over trials that incorrectly specify the unit of analysis are problematic, we will include these results in analyses without adjustment because results suffering from such errors tend to be more conservative than the correct analyses.
- Repeated observations on participants
  o If data from multiple follow‐up rounds are reported, we will choose the follow‐up results that are furthest in time from the time of implementation.
- Events that may reoccur
  o Study results that may be subject to counting events rather than participants, when patients should be the unit of analysis, and do not report on data of total number of patients which have experienced an event, will be excluded.
- Multiple treatment attempts
  o Studies for which the number of attempts at an intervention rather than the unit of randomization is used to produce results will use the same method as cluster randomized trials to correct for potential error.
- Multiple intervention groups
  o Studies with multiple intervention groups that received distinctly different exposures/interventions will report the results of the study arms as separate studies.

#### 3.4.2 Criteria for determination of independent findings

In cases where a singular study provides results on more than one of our outcomes of interest, we will present each result separately. Similarly, if a singular study presents several measures for the same outcome, all results will be presented for completeness, but will be grouped by comparability of the effect size to other effect sizes, as categorized above. In addition, where multiple results for the same outcome of interest measured in the same way are presented within the same paper but are the result of different models/subgroup analyses, one effect size will be reported based on:

- the authors' indication that the effect is one of the primary results of the study, or
- the comparability of the effect size to other studies in the same group, as categorized using the characteristics listed above.

If multiple papers are identified that report on the results from the same intervention/exposure, draw from the same study population and report on the same or very similar outcomes, we will carefully consider which article to use to report effects, conferring with authors or with other methodological experts as necessary, and document efforts to solve inconsistencies across articles. In each case, we will only report results that include our intervention/exposure and outcomes listed above.

#### 3.4.3 Meta‐analysis of primary outcomes

Primary analyses will focus on the primary outcomes listed above to analyze the effects of a given education intervention/exposure on a given outcome. For each study, we will assess whether any effect sizes are independent, dependent, or are the result of clustered analyses. If there are more than two studies grouped by the criterion above and all effect sizes within each group are assessed to be independent (e.g., not drawn from the same sample, not comparing multiple treatments with a common control, etc.), we will conduct a bivariate meta‐analysis using the metareg command in Stata 15 (Harbord & Higgins, 2009; Valentine et al., 2010). A method‐of‐moments random effects meta‐analysis will be used for these results, which is a generalization of the DerSimonian and Laird method (1986). If there are at least 10 studies in a given group and there exist effect sizes that are dependent (e.g., drawn from the same sample), we will run a bivariate random effects meta‐analysis with a robust variance estimator (Tanner‐Smith & Tipton, 2014). Further, if there are at least 10 studies in a given group and there is clustering present in any given study's analysis, we will run a bivariate hierarchical meta‐analysis with a robust variance estimator (Tanner‐Smith & Tipton, 2014). Meta‐analyses with dependent and clustered effect sizes will be run using the robumeta command in Stata 15 (Hedberg, 2014). Measures of between‐study variability will be reported in the form of the *Q*‐statistic, *I* ^2^ and *τ* ^2^. We will use forest plots to display study‐level and overall effect sizes.

#### 3.4.4 Meta‐analysis of secondary outcomes

Not applicable.

#### 3.4.5 Narrative synthesis—Certainty of evidence

In addition to meta‐analysis, we will conduct a narrative synthesis including the GRADE approach adapted for narrative syntheses (Murad et al., 2017). This approach simultaneously considers several factors, including methodological limitations of the studies, how directly the study measured each type of intervention, imprecision, how consistent the findings were across studies, publication bias, and size and direction of effect. The GRADE approach will allow us to indicate the certainty of the evidence.

#### 3.4.6 Causal mechanisms/pathways

This review is primarily focused on understanding the mechanisms linking education to three health outcomes, as laid out in the conceptual framework. The focus of the review will be on the causal effects of education (Box A in conceptual framework) on knowledge, attitudes, resources, and agency (Box B) and on health behaviors and harmful practices (Box C). The recent review noted previously (B. S. Mensch et al., 2019; Psaki et al., 2019) focused on the causal relationships between education (Box A) and health outcomes (Boxes D and E). Therefore, in our data extraction process we will note whether included papers provide estimates of effects on outcomes in Boxes D and E, but these will not be primary or secondary outcomes for this review.

#### 3.4.7 Moderator analyses

An explanation of results of any meta‐analyses will attempt to account for the differences between studies, including the frequency and duration of exposure. Thus, provided there are more than two effect sizes and there is a sufficient amount of heterogeneity for a given moderator, we will attempt to conduct moderator analyses with robust variance estimators that will include the following variables in meta‐regressions:

Study‐level variables:

- Journal articles versus non‐journal articles
- Time of exposure, for example, before/after 1990

Moderator analyses will also utilize method‐of‐moments random effects regressions using the metareg command in Stata 15 (Harbord & Higgins, 2009). Study data used in moderator analyses will be analyzed using the intervention/exposure and outcome grouping framework stated above.

#### 3.4.8 Sensitivity analyses

As a part of sensitivity analyses, REML random effects model results of all applicable meta‐analyses and meta‐regressions will also be presented to assess whether results hold under a different calculation of the between‐study estimation component of *τ* ^2^ (Veroniki et al., 2016). We will also conduct sensitivity analyses to account for study quality.

#### 3.4.9 Assessment of publication and selection bias

To assess the extent to which there may be publication and selection bias present, given enough studies per intervention/exposure and outcome, we will include:

1. A funnel plot, noting possible reasons for asymmetry if it is present, and
2. The trim‐and‐fill procedure to adjust the mean effect size.
3. A meta‐regression using robust variance estimators with standard errors to evaluate small study bias

#### 3.4.10 Treatment of qualitative research

We do not plan to include purely qualitative research in our results, although qualitative research has informed the development of our conceptual framework for this study.

## ROLES AND RESPONSIBILITIES

- Content: Fatima Zahra, Nicole Haberland, Stephanie Psaki
- Systematic review methods: Fatima Zahra, Nicole Haberland, Stephanie Psaki,
- Statistical analysis: TBD
- Information retrieval: Mark Engelbert, 3ie; Erin Eldermire, Cornell; Mary Chu, Population Council
- Advisory group members: Sarah Baird, George Washington University (*confirmed*); Parfait Eloundou‐Enyegue, Cornell University (*confirmed*); Robert Black, Johns Hopkins University (*confirmed*); Julia Behrman, Northwestern University (*confirmed*); Sanyukta Mathur, Population Council, USA (*confirmed*); David Evans, Center for Global Development (*invited*); Caroline Kabiru, APHRC (*confirmed*); Karen Austrian, Population Council Kenya (*confirmed*); Neelanjana Pandey, Population Council India (*confirmed*); Ruth Levine, IDInsight (*confirmed*).

## CONFLICTS OF INTEREST

The review team has no known conflicts of interest.

## PRELIMINARY TIMEFRAME

• Date you plan to submit a draft protocol: May 15, 2021
• Date you plan to submit a draft review: May 1, 2023.

## Supporting information

Supporting information.

## Footnotes

1 There are other health outcomes, such as cardiovascular disease, that may benefit from education and for which other pathways exist (e.g., education may lead to better diet which reduces heart attacks), but these are beyond the scope of this review.

2 Endogeneity arises when an explanatory variable is correlated with the error term either because it is unobserved/unmeasured, or because there is measurement error, or because there is simultaneity, that is, it is jointly determined with the dependent variable (Wooldridge, 2012).

3 The list is drawn from Shadish et al. (2002).

4 Studies that analyze the effects of a RCT but only report data collected after the intervention was implemented were eligible only if they satisfied one of the following criteria: (1) studies do not suffer from issues of statistical power as given by sample size calculations reported in the study, or (2) they report a total sample size of over 10,000, or have more than 1000 observations per cluster in each arm on average. These criteria are roughly drawn from Muralidharan (2017).

5 It is possible that there are effect sizes for which the equations provided may not be sufficient for conversion. In such cases, we will seek the guidance of a meta‐analysis methods expert to assist us in identifying the appropriate conversion equation(s).

6 For OLS and 2SLS models with continuous dependent variables, we strongly prefer using Equations 1.1–1.3, as we recognize that these are more direct estimates of partial correlations. However, in the absence of information from either the articles or from study author(s), we will use Equation 1.4.

7 For example, we will not use the same conversion equation for the results of an OLS models with a continuous independent variable and a continuous dependent variable and those from a logit or probit model.
