# Supplementary material for: PROTOCOL: Causal mechanisms linking education with fertility, HIV, and child mortality: A systematic review
Source: Campbell Syst Rev. 2022 Jun 10;18(2):e1250. doi: 10.1002/cl2.1250 (PMC9187904; doi:10.1002/cl2.1250)
Supplement: Supplementary file 1 — Supporting information. [file CL2-18-e1250-s001.docx]

ppendices

### Section A. Search Strategy and Search Terms

1. Search Strategy

We will use the following search strategy to search ERIC and adapt it for the databases listed above. Searches will be limited to 1990-2022 publication dates. The search terms below are grouped by:

• Geographic Set (S1)

• Education Set (S2)

• Study Set (S5)

• Health and Attitudes Set (S9)

• Year Published Set (S11)

2. Search Terms

| **S#** | **Set Name** | **Search Query** | **Results** |
| --- | --- | --- | --- |
| S1 | LOW- AND MIDDLE-INCOME REGIONS, COUNTRIES AND TEXT WORDS | TI (afghanistan or albania or algeria or "american samoa" or angola or "antigua and barbuda" or antigua or barbuda or argentina or armenia or armenian or aruba or azerbaijan or bahrain or bangladesh or barbados or "republic of belarus" or belarus or byelarus or belorussia or byelorussian or belize or "british honduras" or benin or dahomey or bhutan or bolivia or "bosnia and herzegovina" or bosnia or herzegovina or botswana or bechuanaland or brazil or brasil or bulgaria or "burkina faso" or "burkina fasso" or "upper volta" or burundi or urundi or "cabo verde" or "cape verde" or cambodia or kampuchea or "khmer republic" or cameroon or cameron or cameroun or "central african republic" or "ubangi shari" or chad or chile or china or colombia or comoros or "comoro islands" or "iles comores" or mayotte or "democratic republic of the congo" or "democratic republic congo" or congo or zaire or "costa rica" or "cote d’ivoire" or "cote d’ ivoire" or "cote divoire" or "cote d ivoire" or "ivory coast" or croatia or cuba or cyprus or "czech republic" or czechoslovakia or djibouti or "french somaliland" or dominica or "dominican republic" or ecuador or egypt or "united arab republic" or "el salvador" or "equatorial guinea" or "spanish guinea" or eritrea or estonia or eswatini or swaziland or ethiopia or fiji or gabon or "gabonese republic" or gambia or "georgia republic" or georgian or ghana or "gold coast" or gibraltar or greece or grenada or guam or guatemala or guinea or "guinea bissau" or guyana or "british guiana" or haiti or hispaniola or honduras or hungary or india or indonesia or timor or iran or iraq or "isle of man" or jamaica or jordan or kazakhstan or kazakh or kenya or "democratic people’s republic of korea" or "republic of korea" or "north korea" or "south korea" or korea or kosovo or kyrgyzstan or kirghizia or kirgizstan or "kyrgyz republic" or kirghiz or laos or "lao pdr" or "lao people's democratic republic" or latvia or lebanon or "lebanese republic" or lesotho or basutoland or liberia or libya or "libyan arab jamahiriya" or lithuania or macau or macao or "republic of north macedonia" or macedonia or madagascar or "malagasy republic" or malawi or nyasaland or malaysia or "malay federation" or "malaya federation" or maldives or "indian ocean islands" or "indian ocean" or mali or malta or micronesia or "federated states of micronesia" or kiribati or "marshall islands" or nauru or "northern mariana islands" or palau or tuvalu or mauritania or mauritius or mexico or moldova or moldovian or mongolia or montenegro or morocco or ifni or mozambique or "portuguese east africa" or myanmar or burma or namibia or nepal or "netherlands antilles" or nicaragua or niger or nigeria or oman or muscat or pakistan or panama or "papua new guinea" or "new guinea" or paraguay or peru or philippines or philipines or phillipines or phillippines or poland or "polish people's republic" or portugal or "portuguese republic" or "puerto rico" or romania or russia or "russian federation" or ussr or "soviet union" or "union of soviet socialist republics" or rwanda or ruanda or samoa or pacific islands or polynesia or "samoan islands" or "navigator island" or "navigator islands" or "sao tome and principe" or "saudi arabia" or senegal or serbia or seychelles or "sierra leone" or slovakia or "slovak republic" or slovenia or melanesia or "solomon island" or "solomon islands" or "norfolk island" or "norfolk islands" or somalia or "south africa" or "south sudan" or "sri lanka" or ceylon or "saint kitts and nevis" or "st. kitts and nevis" or "saint lucia" or "st. lucia" or "saint vincent and the grenadines" or "saint vincent" or "st. vincent" or grenadines or sudan or suriname or surinam or "dutch guiana" or "netherlands guiana" or syria or "syrian arab republic" or tajikistan or tadjikistan or tadzhikistan or tadzhik or tanzania or tanganyika or thailand or siam or "timor leste" or "east timor" or togo or "togolese republic" or tonga or "trinidad and tobago" or trinidad or tobago or tunisia or turkey or turkmenistan or turkmen or uganda or ukraine or uruguay or uzbekistan or uzbek or vanuatu or "new hebrides" or venezuela or vietnam or "viet nam" or "middle eas"t or "west bank" or gaza or palestine or yemen or yugoslavia or zambia or zimbabwe or "northern rhodesia" or "global south" or "africa south of the sahara" or "sub-saharan africa" or "subsaharan africa" or "central africa" or "north africa" or "northern africa" or magreb or maghrib or sahara or "southern africa" or "east africa" or "eastern africa" or "west africa" or "western africa" or "west indies" or "indian ocean islands" or caribbean or "central america" or "latin america" or "south and central america" or "south america" or "central asia" or "north asia" or "northern asia" or "southeastern asia" or "south eastern asia" or "southeast asia" or "south east asia" or "western asia" or "east europe" or "eastern europe" or "developing country" or "developing countries" or "developing nation" or "developing nations" or "developing population" or "developing populations" or "developing world" or "less developed countr*" or "less developed nation*" or "less developed population*" or "less developed world" or "lesser developed countr*" or "lesser developed nation*" or "lesser developed population*" or "lesser developed world" or "under developed countr*" or "under developed nation*" or "under developed population*" or "under developed world" or "underdeveloped countr*" or "underdeveloped nation*" or "underdeveloped population*" or "underdeveloped world" or "middle income countr*" or "middle income nation*" or "middle income population*" or "low income countr*" or "low income nation*" or "low income population*" or "lower income countr*" or "lower income nation*" or "lower income population*" or "underserved countr*" or "underserved nation*" or "underserved population*" or "underserved world" or "under served countr*" or "under served nation*" or "under served population*" or "under served world" or "deprived countr*" or "deprived nation*" or "deprived population*" or "deprived world" or "poor countr*" or "poor nation*" or "poor population*" or "poor world" or "poorer countr*" or "poorer nation*" or "poorer population*" or "poorer world" or "developing econom*" or "less developed econom*" or "lesser developed econom*" or "under developed econom*" or "underdeveloped econom*" or "middle income econom*" or "low income econom*" or "lower income econom*" or "low gdp" or "low gnp" or "low gross domestic" or "low gross national" or "lower gdp" or "lower gnp" or "lower gross domestic" or "lower gross national" or lmic or lmics or "third world" or "lami countr*" or "transitional countr*" or "emerging econom*" or "emerging nation*") OR AB (afghanistan or albania or algeria or "american samoa" or angola or "antigua and barbuda" or antigua or barbuda or argentina or armenia or armenian or aruba or azerbaijan or bahrain or bangladesh or barbados or "republic of belarus" or belarus or byelarus or belorussia or byelorussian or belize or "british honduras" or benin or dahomey or bhutan or bolivia or "bosnia and herzegovina" or bosnia or herzegovina or botswana or bechuanaland or brazil or brasil or bulgaria or "burkina faso" or "burkina fasso" or "upper volta" or burundi or urundi or "cabo verde" or "cape verde" or cambodia or kampuchea or "khmer republic" or cameroon or cameron or cameroun or "central african republic" or "ubangi shari" or chad or chile or china or colombia or comoros or "comoro islands" or "iles comores" or mayotte or "democratic republic of the congo" or "democratic republic congo" or congo or zaire or "costa rica" or "cote d’ivoire" or "cote d’ ivoire" or "cote divoire" or "cote d ivoire" or "ivory coast" or croatia or cuba or cyprus or "czech republic" or czechoslovakia or djibouti or "french somaliland" or dominica or "dominican republic" or ecuador or egypt or "united arab republic" or "el salvador" or "equatorial guinea" or "spanish guinea" or eritrea or estonia or eswatini or swaziland or ethiopia or fiji or gabon or "gabonese republic" or gambia or "georgia republic" or georgian or ghana or "gold coast" or gibraltar or greece or grenada or guam or guatemala or guinea or "guinea bissau" or guyana or "british guiana" or haiti or hispaniola or honduras or hungary or india or indonesia or timor or iran or iraq or "isle of man" or jamaica or jordan or kazakhstan or kazakh or kenya or "democratic people’s republic of korea" or "republic of korea" or "north korea" or "south korea" or korea or kosovo or kyrgyzstan or kirghizia or kirgizstan or "kyrgyz republic" or kirghiz or laos or "lao pdr" or "lao people's democratic republic" or latvia or lebanon or "lebanese republic" or lesotho or basutoland or liberia or libya or "libyan arab jamahiriya" or lithuania or macau or macao or "republic of north macedonia" or macedonia or madagascar or "malagasy republic" or malawi or nyasaland or malaysia or "malay federation" or "malaya federation" or maldives or "indian ocean islands" or "indian ocean" or mali or malta or micronesia or "federated states of micronesia" or kiribati or "marshall islands" or nauru or "northern mariana islands" or palau or tuvalu or mauritania or mauritius or mexico or moldova or moldovian or mongolia or montenegro or morocco or ifni or mozambique or "portuguese east africa" or myanmar or burma or namibia or nepal or "netherlands antilles" or nicaragua or niger or nigeria or oman or muscat or pakistan or panama or "papua new guinea" or "new guinea" or paraguay or peru or philippines or philipines or phillipines or phillippines or poland or "polish people's republic" or portugal or "portuguese republic" or "puerto rico" or romania or russia or "russian federation" or ussr or "soviet union" or "union of soviet socialist republics" or rwanda or ruanda or samoa or pacific islands or polynesia or "samoan islands" or "navigator island" or "navigator islands" or "sao tome and principe" or "saudi arabia" or senegal or serbia or seychelles or "sierra leone" or slovakia or "slovak republic" or slovenia or melanesia or "solomon island" or "solomon islands" or "norfolk island" or "norfolk islands" or somalia or "south africa" or "south sudan" or "sri lanka" or ceylon or "saint kitts and nevis" or "st. kitts and nevis" or "saint lucia" or "st. lucia" or "saint vincent and the grenadines" or "saint vincent" or "st. vincent" or grenadines or sudan or suriname or surinam or "dutch guiana" or "netherlands guiana" or syria or "syrian arab republic" or tajikistan or tadjikistan or tadzhikistan or tadzhik or tanzania or tanganyika or thailand or siam or "timor leste" or "east timor" or togo or "togolese republic" or tonga or "trinidad and tobago" or trinidad or tobago or tunisia or turkey or turkmenistan or turkmen or uganda or ukraine or uruguay or uzbekistan or uzbek or vanuatu or "new hebrides" or venezuela or vietnam or "viet nam" or "middle eas"t or "west bank" or gaza or palestine or yemen or yugoslavia or zambia or zimbabwe or "northern rhodesia" or "global south" or "africa south of the sahara" or "sub-saharan africa" or "subsaharan africa" or "central africa" or "north africa" or "northern africa" or magreb or maghrib or sahara or "southern africa" or "east africa" or "eastern africa" or "west africa" or "western africa" or "west indies" or "indian ocean islands" or caribbean or "central america" or "latin america" or "south and central america" or "south america" or "central asia" or "north asia" or "northern asia" or "southeastern asia" or "south eastern asia" or "southeast asia" or "south east asia" or "western asia" or "east europe" or "eastern europe" or "developing country" or "developing countries" or "developing nation" or "developing nations" or "developing population" or "developing populations" or "developing world" or "less developed countr*" or "less developed nation*" or "less developed population*" or "less developed world" or "lesser developed countr*" or "lesser developed nation*" or "lesser developed population*" or "lesser developed world" or "under developed countr*" or "under developed nation*" or "under developed population*" or "under developed world" or "underdeveloped countr*" or "underdeveloped nation*" or "underdeveloped population*" or "underdeveloped world" or "middle income countr*" or "middle income nation*" or "middle income population*" or "low income countr*" or "low income nation*" or "low income population*" or "lower income countr*" or "lower income nation*" or "lower income population*" or "underserved countr*" or "underserved nation*" or "underserved population*" or "underserved world" or "under served countr*" or "under served nation*" or "under served population*" or "under served world" or "deprived countr*" or "deprived nation*" or "deprived population*" or "deprived world" or "poor countr*" or "poor nation*" or "poor population*" or "poor world" or "poorer countr*" or "poorer nation*" or "poorer population*" or "poorer world" or "developing econom*" or "less developed econom*" or "lesser developed econom*" or "under developed econom*" or "underdeveloped econom*" or "middle income econom*" or "low income econom*" or "lower income econom*" or "low gdp" or "low gnp" or "low gross domestic" or "low gross national" or "lower gdp" or "lower gnp" or "lower gross domestic" or "lower gross national" or lmic or lmics or "third world" or "lami countr*" or "transitional countr*" or "emerging econom*" or "emerging nation*") OR KW (afghanistan or albania or algeria or "american samoa" or angola or "antigua and barbuda" or antigua or barbuda or argentina or armenia or armenian or aruba or azerbaijan or bahrain or bangladesh or barbados or "republic of belarus" or belarus or byelarus or belorussia or byelorussian or belize or "british honduras" or benin or dahomey or bhutan or bolivia or "bosnia and herzegovina" or bosnia or herzegovina or botswana or bechuanaland or brazil or brasil or bulgaria or "burkina faso" or "burkina fasso" or "upper volta" or burundi or urundi or "cabo verde" or "cape verde" or cambodia or kampuchea or "khmer republic" or cameroon or cameron or cameroun or "central african republic" or "ubangi shari" or chad or chile or china or colombia or comoros or "comoro islands" or "iles comores" or mayotte or "democratic republic of the congo" or "democratic republic congo" or congo or zaire or "costa rica" or "cote d’ivoire" or "cote d’ ivoire" or "cote divoire" or "cote d ivoire" or "ivory coast" or croatia or cuba or cyprus or "czech republic" or czechoslovakia or djibouti or "french somaliland" or dominica or "dominican republic" or ecuador or egypt or "united arab republic" or "el salvador" or "equatorial guinea" or "spanish guinea" or eritrea or estonia or eswatini or swaziland or ethiopia or fiji or gabon or "gabonese republic" or gambia or "georgia republic" or georgian or ghana or "gold coast" or gibraltar or greece or grenada or guam or guatemala or guinea or "guinea bissau" or guyana or "british guiana" or haiti or hispaniola or honduras or hungary or india or indonesia or timor or iran or iraq or "isle of man" or jamaica or jordan or kazakhstan or kazakh or kenya or "democratic people’s republic of korea" or "republic of korea" or "north korea" or "south korea" or korea or kosovo or kyrgyzstan or kirghizia or kirgizstan or "kyrgyz republic" or kirghiz or laos or "lao pdr" or "lao people's democratic republic" or latvia or lebanon or "lebanese republic" or lesotho or basutoland or liberia or libya or "libyan arab jamahiriya" or lithuania or macau or macao or "republic of north macedonia" or macedonia or madagascar or "malagasy republic" or malawi or nyasaland or malaysia or "malay federation" or "malaya federation" or maldives or "indian ocean islands" or "indian ocean" or mali or malta or micronesia or "federated states of micronesia" or kiribati or "marshall islands" or nauru or "northern mariana islands" or palau or tuvalu or mauritania or mauritius or mexico or moldova or moldovian or mongolia or montenegro or morocco or ifni or mozambique or "portuguese east africa" or myanmar or burma or namibia or nepal or "netherlands antilles" or nicaragua or niger or nigeria or oman or muscat or pakistan or panama or "papua new guinea" or "new guinea" or paraguay or peru or philippines or philipines or phillipines or phillippines or poland or "polish people's republic" or portugal or "portuguese republic" or "puerto rico" or romania or russia or "russian federation" or ussr or "soviet union" or "union of soviet socialist republics" or rwanda or ruanda or samoa or pacific islands or polynesia or "samoan islands" or "navigator island" or "navigator islands" or "sao tome and principe" or "saudi arabia" or senegal or serbia or seychelles or "sierra leone" or slovakia or "slovak republic" or slovenia or melanesia or "solomon island" or "solomon islands" or "norfolk island" or "norfolk islands" or somalia or "south africa" or "south sudan" or "sri lanka" or ceylon or "saint kitts and nevis" or "st. kitts and nevis" or "saint lucia" or "st. lucia" or "saint vincent and the grenadines" or "saint vincent" or "st. vincent" or grenadines or sudan or suriname or surinam or "dutch guiana" or "netherlands guiana" or syria or "syrian arab republic" or tajikistan or tadjikistan or tadzhikistan or tadzhik or tanzania or tanganyika or thailand or siam or "timor leste" or "east timor" or togo or "togolese republic" or tonga or "trinidad and tobago" or trinidad or tobago or tunisia or turkey or turkmenistan or turkmen or uganda or ukraine or uruguay or uzbekistan or uzbek or vanuatu or "new hebrides" or venezuela or vietnam or "viet nam" or "middle eas"t or "west bank" or gaza or palestine or yemen or yugoslavia or zambia or zimbabwe or "northern rhodesia" or "global south" or "africa south of the sahara" or "sub-saharan africa" or "subsaharan africa" or "central africa" or "north africa" or "northern africa" or magreb or maghrib or sahara or "southern africa" or "east africa" or "eastern africa" or "west africa" or "western africa" or "west indies" or "indian ocean islands" or caribbean or "central america" or "latin america" or "south and central america" or "south america" or "central asia" or "north asia" or "northern asia" or "southeastern asia" or "south eastern asia" or "southeast asia" or "south east asia" or "western asia" or "east europe" or "eastern europe" or "developing country" or "developing countries" or "developing nation" or "developing nations" or "developing population" or "developing populations" or "developing world" or "less developed countr*" or "less developed nation*" or "less developed population*" or "less developed world" or "lesser developed countr*" or "lesser developed nation*" or "lesser developed population*" or "lesser developed world" or "under developed countr*" or "under developed nation*" or "under developed population*" or "under developed world" or "underdeveloped countr*" or "underdeveloped nation*" or "underdeveloped population*" or "underdeveloped world" or "middle income countr*" or "middle income nation*" or "middle income population*" or "low income countr*" or "low income nation*" or "low income population*" or "lower income countr*" or "lower income nation*" or "lower income population*" or "underserved countr*" or "underserved nation*" or "underserved population*" or "underserved world" or "under served countr*" or "under served nation*" or "under served population*" or "under served world" or "deprived countr*" or "deprived nation*" or "deprived population*" or "deprived world" or "poor countr*" or "poor nation*" or "poor population*" or "poor world" or "poorer countr*" or "poorer nation*" or "poorer population*" or "poorer world" or "developing econom*" or "less developed econom*" or "lesser developed econom*" or "under developed econom*" or "underdeveloped econom*" or "middle income econom*" or "low income econom*" or "lower income econom*" or "low gdp" or "low gnp" or "low gross domestic" or "low gross national" or "lower gdp" or "lower gnp" or "lower gross domestic" or "lower gross national" or lmic or lmics or "third world" or "lami countr*" or "transitional countr*" or "emerging econom*" or "emerging nation*") | 137,213 |
| S2 | EDUCATION | TI (education* OR school* OR learn* OR "grade attainment" OR literac* OR literate OR numeracy OR numerate OR textbook* OR "cognitive abilit*" OR (score* N3 test*) OR (exam* N3 tak*) OR curricul* OR "sanitary facilit*" OR "student costs" OR (academic W1 (achieve* OR engag*)) OR "enrollment status" OR "enrolment status" OR (barrier* N3 (retention OR retain)) OR (teacher* N3 (bias* OR attitude*))) OR AB (education* OR school* OR learn* OR "grade attainment" OR literac* OR literate OR numeracy OR numerate OR textbook* OR "cognitive abilit*" OR (score* N3 test*) OR (exam* N3 tak*) OR curricul* OR "sanitary facilit*" OR "student costs" OR (academic W1 (achieve* OR engag*)) OR "enrollment status" OR "enrolment status" OR (barrier* N3 (retention OR retain)) OR (teacher* N3 (bias* OR attitude*))) OR KW (education* OR school* OR learn* OR "grade attainment" OR literac* OR literate OR numeracy OR numerate OR textbook* OR "cognitive abilit*" OR (score* N3 test*) OR (exam* N3 tak*) OR curricul* OR "sanitary facilit*" OR "student costs" OR (academic W1 (achieve* OR engag*)) OR "enrollment status" OR "enrolment status" OR (barrier* N3 (retention OR retain)) OR (teacher* N3 (bias* OR attitude*))) | 1,212,033 |
| S3 | STUDY DESIGN | TI ("randomized evaluation*" OR "random* control* trial" OR (control* N3 endogeneity) OR (regression W1 discontinuit*) OR "instrumental variable*" OR "interrupted time series" OR (pre W1 test*) OR (post W1 test*) OR pretest* OR posttest* OR "match* comparison group*" OR (matching N2 procedure*) OR ((comparison OR control) W1 group*) OR quasiexperiment* OR quasi-experiment* OR "mixed method*" OR (mediat* N3 analy*) OR "path* analys*") OR AB ("randomized evaluation*" OR random* control* trial" OR (control* N3 endogeneity) OR (regression W1 discontinuit*) OR "instrumental variable*" OR "interrupted time series" OR (pre W1 test*) OR (post W1 test*) OR pretest* OR posttest* OR "match* comparison group*" OR (matching N2 procedure*) OR ((comparison OR control) W1 group*) OR quasiexperiment* OR quasi-experiment* OR "mixed method*" OR (mediat* N3 analy*) OR "path* analys*") OR KW ("randomized evaluation*" OR "random* control* trial" OR (control* N3 endogeneity) OR (regression W1 discontinuit*) OR "instrumental variable*" OR "interrupted time series" OR (pre W1 test*) OR (post W1 test*) OR pretest* OR posttest* OR "match* comparison group*" OR (matching N2 procedure*) OR ((comparison OR control) W1 group*) OR quasiexperiment* OR quasi-experiment* OR "mixed method*" OR (mediat* N3 analy*) OR "path* analys*") | 4,342 |
| S4 | STUDY RESULT | TI (((intervention* OR program* OR policy OR policies OR policymakers OR legislation) N4 (evidence OR evaluat* OR effect* OR result*)) OR "outcome* measur*" OR causal OR counterfactual OR attribut* OR "case stud*" OR "best practice*" OR "what works" OR mechanism*) OR AB (((intervention* OR program* OR policy OR policies OR policymakers OR legislation) N4 (evidence OR evaluat* OR effect* OR result*)) OR "outcome* measur*" OR causal OR counterfactual OR attribut* OR "case stud*" OR "best practice*" OR "what works" OR mechanism*) OR KW (((intervention* OR program* OR policy OR policies OR policymakers OR legislation) N4 (evidence OR evaluat* OR effect* OR result*)) OR "outcome* measur*" OR causal OR counterfactual OR attribut* OR "case stud*" OR "best practice*" OR "what works" OR mechanism*) | 227,179 |
| S5 | STUDY SET | S3 OR S4 | 230,089 |
| S6 | MATERNAL AND NEONATAL HEALTH | TI (fertility OR nullipar* OR primipar* OR multipar* OR "number of children" OR "age at marriage" OR "age at first birth" OR "age at first pregnancy" OR "reproductive health" OR STI OR STD OR ("sexually transmitted" W1 (disease OR infection)) OR HIV OR HIV/AIDS OR HIV-AIDS OR syphilis OR gonorrhea OR gonorrhoea OR ((maternal OR mother*) N3 (illness* OR sickness* OR disease* OR ill-health)) OR ((child* OR infant OR infants OR neonat*) N3 (mortal* OR death* OR fatalit*))) OR AB (fertility OR nullipar* OR primipar* OR multipar* OR "number of children" OR "age at marriage" OR "age at first birth" OR "age at first pregnancy" OR "reproductive health" OR STI OR STD OR ("sexually transmitted" W1 (disease OR infection)) OR HIV OR HIV/AIDS OR HIV-AIDS OR syphilis OR gonorrhea OR gonorrhoea OR ((maternal OR mother*) N3 (illness* OR sickness* OR disease* OR ill-health)) OR ((child* OR infant OR infants OR neonat*) N3 (mortal* OR death* OR fatalit*))) OR KW (fertility OR nullipar* OR primipar* OR multipar* OR "number of children" OR "age at marriage" OR "age at first birth" OR "age at first pregnancy" OR "reproductive health" OR STI OR STD OR ("sexually transmitted" W1 (disease OR infection)) OR HIV OR HIV/AIDS OR HIV-AIDS OR syphilis OR gonorrhea OR gonorrhoea OR ((maternal OR mother*) N3 (illness* OR sickness* OR disease* OR ill-health)) OR ((child* OR infant OR infants OR neonat*) N3 (mortal* OR death* OR fatalit*))) | 9,850 |
| S7 | KNOWLEDGE, ATTITUDES, RESOURCES, AGENCY | TI ((Household N3 (income OR asset* OR saving*)) OR ((partner OR partners OR spous* OR husband* OR wife OR wives OR parent* OR maternal OR paternal) N3 (education OR income)) OR ((attitud* OR knowledge OR prefer* OR desire* OR aspiration*) N4 (contracept* OR "birth control" OR gender OR violence OR marriage OR career)) OR ((woman OR women OR gender OR household OR marital OR spousal OR relationship*) N4 ("decision making" OR empowerment OR agency OR power OR autonomy OR mobility OR "freedom of movement" OR ((control OR negotiat* OR power) N4 (resource* OR spend* OR money OR income)))) OR (condom N4 (negotiat* OR decision*))) OR AB ((Household N3 (income OR asset* OR saving*)) OR ((partner OR partners OR spous* OR husband* OR wife OR wives OR parent* OR maternal OR paternal) N3 (education OR income)) OR ((attitud* OR knowledge OR prefer* OR desire* OR aspiration*) N4 (contracept* OR "birth control" OR gender OR violence OR marriage OR career)) OR ((woman OR women OR gender OR household OR marital OR spousal OR relationship*) N4 ("decision making" OR empowerment OR agency OR power OR autonomy OR mobility OR "freedom of movement" OR ((control OR negotiat* OR power) N4 (resource* OR spend* OR money OR income)))) OR (condom N4 (negotiat* OR decision*))) OR KW ((Household N3 (income OR asset* OR saving*)) OR ((partner OR partners OR spous* OR husband* OR wife OR wives OR parent* OR maternal OR paternal) N3 (education OR income)) OR ((attitud* OR knowledge OR prefer* OR desire* OR aspiration*) N4 (contracept* OR "birth control" OR gender OR violence OR marriage OR career)) OR ((woman OR women OR gender OR household OR marital OR spousal OR relationship*) N4 ("decision making" OR empowerment OR agency OR power OR autonomy OR mobility OR "freedom of movement" OR ((control OR negotiat* OR power) N4 (resource* OR spend* OR money OR income)))) OR (condom N4 (negotiat* OR decision*))) | 28,042 |
| S8 | HEALTH BEHAVIORS and HARMFUL PRACTICES | TI (((contracept* OR condom OR prophylact*) N3 (modern OR use OR usage OR inject*)) OR "sexual behavior" OR "sexual behaviour" OR "sexual partner*" OR "transactional sex" OR "unprotected sex" OR "risky sex" OR "high risk sex" OR "high-risk sex" OR ((antenatal OR prenatal OR pregnan* OR maternal) N4 (care OR visit*)) OR (birth N3 ((skill* N3 attend*) OR hospital OR hospitals OR "health center*")) OR hygiene OR sanitation OR handwash* OR (wash* N2 hand*) OR "clean water" OR soap OR (child* N4 (illness* OR "health care" OR immunization OR immunisation OR immunize OR immunise OR vaccin* OR nutrition OR stunt* OR weight OR height OR wasted OR wasting)) OR breastfeed* OR breastfed OR parenting OR "parent time" OR (("intimate partner" OR domestic OR spousal OR child* OR physical OR sexual OR emotional) N4 (violence OR abuse OR neglect)) OR ((child* OR adolescent* OR early) N3 marriage)) OR AB (((contracept* OR condom OR prophylact*) N3 (modern OR use OR usage OR inject*)) OR "sexual behavior" OR "sexual behaviour" OR "sexual partner*" OR "transactional sex" OR "unprotected sex" OR "risky sex" OR "high risk sex" OR "high-risk sex" OR ((antenatal OR prenatal OR pregnan* OR maternal) N4 (care OR visit*)) OR (birth N3 ((skill* N3 attend*) OR hospital OR hospitals OR "health center*")) OR hygiene OR sanitation OR handwash* OR (wash* N2 hand*) OR "clean water" OR soap OR (child* N4 (illness* OR "health care" OR immunization OR immunisation OR immunize OR immunise OR vaccin* OR nutrition OR stunt* OR weight OR height OR wasted OR wasting)) OR breastfeed* OR breastfed OR parenting OR "parent time" OR (("intimate partner" OR domestic OR spousal OR child* OR physical OR sexual OR emotional) N4 (violence OR abuse OR neglect)) OR ((child* OR adolescent* OR early) N3 marriage)) OR KW (((contracept* OR condom OR prophylact*) N3 (modern OR use OR usage OR inject*)) OR "sexual behavior" OR "sexual behaviour" OR "sexual partner*" OR "transactional sex" OR "unprotected sex" OR "risky sex" OR "high risk sex" OR "high-risk sex" OR ((antenatal OR prenatal OR pregnan* OR maternal) N4 (care OR visit*)) OR (birth N3 ((skill* N3 attend*) OR hospital OR hospitals OR "health center*")) OR hygiene OR sanitation OR handwash* OR (wash* N2 hand*) OR "clean water" OR soap OR (child* N4 (illness* OR "health care" OR immunization OR immunisation OR immunize OR immunise OR vaccin* OR nutrition OR stunt* OR weight OR height OR wasted OR wasting)) OR breastfeed* OR breastfed OR parenting OR "parent time" OR (("intimate partner" OR domestic OR spousal OR child* OR physical OR sexual OR emotional) N4 (violence OR abuse OR neglect)) OR ((child* OR adolescent* OR early) N3 marriage)) | 30,222 |
| S9 | HEALTH AND ATTITUDES | S6 OR S7 OR S8 | 62,882 |
| S10 | LMICs + EDUCATION + STUDY DESIGN + HEALTH | S1 AND S2 AND S5 AND S9 | 896 |
| S11 | YEAR PUBLISHED | 1990 - present | 803 |

### Section B. Assessment of Risk of Bias

The following risk of bias tools were adapted from RoB 2 (Higgins et al., 2018) for randomized studies, and ROBINS-I (Sterne et al., 2016) and Psaki et al., (2019) for non-randomized studies. The following are the values for response options:

- Y = “Yes”
- PY = “Possibly Yes”
- PN = “Possibly No”
- N = “No”
- NI = “No Information”
- NA = “Not Applicable”

Values in green indicate response options for which there may be low risk of bias. Values in red indicate response options for which there may be high risk of bias. We follow the guidelines for RoB 2, ROBINS-I and Psaki et al., (2019) as closely as possible to judge for risk of bias.

**1) Randomized Studies**

| **Bias Domain** | **Signalling questions** | **Response Options** |
| --- | --- | --- |
| **Bias from randomization process** | 1.1 Was the allocation sequence random? | Y / PY / PN / N / NI |
|  | 1.2 Was the allocation sequence concealed until participants were enrolled and assigned to interventions? | Y / PY / PN / N / NI |
|  | 1.3 Did baseline differences between intervention groups suggest a problem with the randomization process? | Y / PY / PN / N / NI |
|  | **Risk-of-bias judgement** | Low / High / Some concerns |
| **Bias from deviations in assignment from intended interventions** | 2.1. Were participants aware of their assigned intervention during the trial? | Y / PY / PN / N / NI |
|  | 2.2. Were carers and people delivering the interventions aware of participants' assigned intervention during the trial? | Y / PY / PN / N / NI |
|  | 2.3. If Y/PY/NI to 2.1 or 2.2, Were there deviations from the intended intervention that arose because of the experimental context? | NA / Y / PY / PN / N / NI |
|  | 2.4. If Y/PY to 2.3, Were these deviations from intended intervention balanced between groups? | NA / Y / PY / PN / N / NI |
|  | 2.5 If N/PN/NI to 2.4, were these deviations likely to have affected the outcome? | NA / Y / PY / PN / N / NI |
|  | 2.6 Was an appropriate analysis used to estimate the effect of assignment to intervention? | Y / PY / PN / N / NI |
|  | 2.7 If N/PN/NI to 2.6, was there potential for a substantial impact (on the result) of the failure to analyse participants in the group to which they were randomized? | NA / Y / PY / PN / N / NI |
|  | **Risk-of-bias judgement** | Low / High / Some concerns |
| **Bias from missing outcome data** | 3.1 Were data for this outcome available for all, or nearly all, participants randomized? | Y / PY / PN / N / NI |
|  | 3.2 If N/PN/NI to 3.1, is there evidence that result was not biased by missing outcome data? | NA / Y / PY / PN / N |
|  | 3.3 If N/PN to 3.2, could missingness in the outcome depend on its true value? | NA / Y / PY / PN / N / NI |
|  | 3.4 If Y/PY/NI to 3.3, do the proportions of missing outcome data differ between intervention groups? | NA / Y / PY / PN / N / NI |
|  | 3.5 If Y/PY/NI to 3.3, is it likely that missingness in the outcome depended on its true value? | NA / Y / PY / PN / N / NI |
|  | **Risk-of-bias judgement** | Low / High / Some concerns |
| **Bias due to measurement of outcome** | 4.1 Was the method of measuring the outcome inappropriate? | Y / PY / PN / N / NI |
|  | 4.2 Could measurement or ascertainment of the outcome have differed between intervention groups? | Y / PY / PN / N / NI |
|  | 4.3 If N/PN/NI to 4.1 and 4.2, were outcome assessors aware of the intervention received by study participants? | Y / PY / PN / N / NI |
|  | 4.4 If Y/PY/NI to 4.3, could assessment of the outcome have been influenced by knowledge of intervention received? | NA / Y / PY / PN / N / NI |
|  | 4.5 If Y/PY/NI to 4.4, is it likely that assessment of the outcome was influenced by knowledge of intervention received? | NA / Y / PY / PN / N / NI |
|  | **Risk-of-bias judgement** | Low / High / Some concerns |
| **Bias due to selection of reported result** | 5.1 Was the trial analysed in accordance with a pre-specified plan that was finalized before unblinded outcome data were available for analysis? | Y / PY / PN / N / NI |
|  | Is the numerical result being assessed likely to have been selected, on the basis of the results, from... |  |
|  | 5.2.... multiple outcome measurements (e.g. scales, definitions, time points) within the outcome domain? | Y / PY / PN / N / NI |
|  | 5.3... multiple analyses of the data? | Y / PY / PN / N / NI |
|  | **Risk-of-bias judgement** | Low / High / Some concerns |
| **Overall risk of bias** | **Risk-of-bias judgement** | Low / High / Some concerns |

**2) Non-randomized Studies**

| **Bias Domain** | **Signalling questions** | **Response Options** |
| --- | --- | --- |
| **Bias due to confounding** | 1.1 Is there potential for confounding of the effect of the intervention in this study? | Y / PY / PN / N / NI |
|  | If Y/PY to 1.1: Assess time-varying confounding: |  |
|  | 1.2 Was the analysis based on splitting participants’ follow-up time according to intervention received?  If N/PN, answer questions relating to baseline confounding (1.4 to 1.6)  If Y/PY, proceed to question 1.3. | NA / Y / PY / PN / N / NI |
|  | 1.3 Were intervention discontinuations or switches likely to be related to factors that are prognostic for the outcome?  If N/PN, answer questions relating to baseline confounding (1.4 to 1.6)  If Y/PY, answer questions relating to both baseline and time-varying confounding (1.7 and 1.8) | NA / Y / PY / PN / N / NI |
|  | **Related to baseline confounding only**: |  |
|  | 1.4 Did the authors use an appropriate analysis method that controlled for all the important confounding domains? | NA / Y / PY / PN / N / NI |
|  | 1.5 If Y/PY to 1.4, were confounding domains that were controlled for measured validly and reliably for measured variables available in this study? | NA / Y / PY / PN / N / NI |
|  | 1.6 Did the authors control for any post-intervention variables that could have been affected by the intervention? | NA / Y / PY / PN / N / NI |
|  | **Related to time-varying confounding**: |  |
|  | 1.7 Did the authors use an appropriate analysis method that adjusted for time-varying confounding? | NA / Y / PY / PN / N / NI |
|  | 1.8 If Y/PY to 1.7, were confounding domains that were adjusted for measured validly and reliably by the variables available in this study? | NA / Y / PY / PN / N / NI |
|  | **Risk-of-bias judgement** | Low / High / Some concerns |
| **Bias in selection of participants into the study** | 2.1 Was selection of participants into the study (or into the analysis) based on participant characteristics observed after the start of the intervention? | Y / PY / PN / N / NI |
|  | If Y/PY to 2.1: |  |
|  | 2.2 Were the post-intervention variables that influenced selection likely to be associated with the intervention? | NA / Y / PY / PN / N / NI |
|  | 2.3 Were the post-interventions that influenced selection likely to be influenced by the outcome or a cause of the outcome? | NA / Y / PY / PN / N / NI |
|  | 2.4 Do start of follow-up and start of intervention coincide for most participants? | Y / PY / PN / N / NI |
|  | 2.5 If Y/PY to 2.2 and 2.3, or N/PN to 2.4, were adjustment techniques used likely to correct for the presence of selection biases? | NA / Y / PY / PN / N / NI |
|  | **Risk-of-bias judgement** | Low / High / Some concerns |
| **Bias in classification of interventions** | 3.1 Were intervention groups clearly defined? | Y / PY / PN / N / NI |
|  | 3.2 Was the information used to define intervention groups recorded at the start of the intervention? | Y / PY / PN / N / NI |
|  | 3.3 Could classification of intervention status have been affected by knowledge of the outcome or risk of the outcome? | Y / PY / PN / N / NI |
|  | **Risk-of-bias judgement** | Low / High / Some concerns |
| **Bias due to deviations from intended interventions** | 4.1 Were the deviations from the intended intervention beyond what would be expected in usual practice? | Y / PY / PN / N / NI |
|  | 4.2 If Y/PY to 4.1, were these deviations from intended intervention unbalanced between groups *and* likely to have affected the outcome? | NA / Y / PY / PN / N / NI |
|  | **Risk-of-bias judgement** | Low / High / Some concerns |
| **Bias due to missing data** | 5.1 Were outcome data available for all, or nearly all, participants? | Y / PY / PN / N / NI |
|  | 5.2 Were participants excluded due to missing data on intervention status? | Y / PY / PN / N / NI |
|  | 5.3 Were participants excluded due to missing data on other variables needed for the analysis? | Y / PY / PN / N / NI |
|  | If PN/N to 5.1, or Y/PY to 5.2 or 5.3: |  |
|  | 5.4 Are the proportion of participants and reasons for missing data similar across interventions? | NA / Y / PY / PN / N / NI |
|  | 5.5 Is there evidence that results were robust to the presence of missing data? | NA / Y / PY / PN / N / NI |
|  | **Risk-of-bias judgement** | Low / High / Some concerns |
| **Bias in measurement of outcomes** | 6.1 Could the outcome measure have been influenced by knowledge of the intervention received? | Y / PY / PN / N / NI |
|  | 6.2 Were outcome assessors aware of the intervention received by study participants? | Y / PY / PN / N / NI |
|  | 6.3 Were the methods of outcome assessment comparable across intervention groups? | Y / PY / PN / N / NI |
|  | 6.4 Were any systematic errors in measurement of the outcome related to intervention received? | Y / PY / PN / N / NI |
|  | **Risk-of-bias judgement** | Low / High / Some concerns |
| **Bias in selection of the reported result** | Is the reported effect estimate likely to be selected on the basis of the results, from… |  |
|  | 7.1 …multiple outcome *measurements* within the outcome domain? | Y / PY / PN / N / NI |
|  | 7.2 …multiple *analyses* of the intervention-outcome relationship? | Y / PY / PN / N / NI |
|  | 7.3 …different subgroups? | Y / PY / PN / N / NI |
|  | **Risk-of-bias judgement** | Low / High / Some concerns |
| **Methods-specific criteria** | **Natural experiments** (Craig et al., 2012): | Y / N |
|  | 8.1.1 The authors note that the analysis was of a natural experiment or an exogenous change/event. | Y / PY / PN / N / NI |
|  | 8.1.2 The context in which the natural experiment occurred was described. | Y / PY / PN / N / NI |
|  | 8.1.3 The intervention and assignment process were described. | Y / PY / PN / N / NI |
|  | 8.1.4 The methods used to estimate impact were explicitly stated. | Y / PY / PN / N / NI |
|  | 8.1.5 Quantitative procedures to reduce the risk of bias were implemented and qualitative and/or theoretical justification was provided for use of aforementioned procedures. | Y / PY / PN / N / NI |
|  | **Regression discontinuity design:** | Y / N |
|  | 8.2 Authors gave justification for the distance from the cut-off point between treatment and control or authors weighted the matches to their distance to the cut-off point. | Y / PY / PN / N / NI |
|  | **Matching methods** (Stuart, 2010): | Y / N |
|  | 8.3.1 Covariates used were explicitly listed and were all not associated with treatment assignment and outcomes of interest. | Y / PY / PN / N / NI |
|  | 8.3.2 Authors performed and reported the results of a Rosenbaum test (or equivalent) for hidden bias. | Y / PY / PN / N / NI |
|  | 8.3.3 More than 90% of matches made. | Y / PY / PN / N / NI |
|  | If the study uses Mahalanobis: |  |
|  | 8.3.4 Fewer than 8 covariates were utilized. | NA / Y / PY / PN / N / NI |
|  | 8.3.5 Authors state that all covariates are normally distributed. | NA / Y / PY / PN / N / NI |
|  | If the study uses propensity score matching: |  |
|  | 8.3.6 Propensity score estimation model has been validated (e.g. logistic regression, boosted CART, generalized boosted models). | NA / Y / PY / PN / N / NI |
|  | 8.3.7 If linear propensity score matching, used a caliper of 0.25 standard deviations of the linear propensity score or justification is given for a larger caliper. | NA / Y / PY / PN / N / NI |
|  | If the study uses nearest-neighbor matching: |  |
|  | 8.3.8 If caliper matching, 90% of matches are within one standard deviation of the mean of covariate, or justification is given for a larger caliper. | NA / Y / PY / PN / N / NI |
|  | 8.3.9 If with replacement, causal inference issues are noted and addressed in the analysis (e.g. using frequency weights). | NA / Y / PY / PN / N / NI |
|  | 8.3.10 If with replacement, the number of times each control is matched is noted and justification is given if there are a large number of matches for each control. | NA / Y / PY / PN / N / NI |
|  | **Heckman correction and instrumental variables:** | Y / N |
|  | 8.4.1 The instrumenting equation is significant at F≥10 or the author reports and assesses whether the R-squared of the instrumenting equation is adequate for appropriate identification. | Y / PY / PN / N / NI |
|  | 8.4.2 All instruments/corrections are reported and significant p≤0.05. | Y / PY / PN / N / NI |
|  | 8.4.3 The authors qualitatively assess the exogeneity of the instrument/identifier and provide a theoretical justification for it. | Y / PY / PN / N / NI |
|  | 8.4.4 A Hausman test for exogeneity (or equivalent) was performed and reported where the authors cannot reject the null at 95% confidence. | Y / PY / PN / N / NI |
|  | **Maximum likelihood models or OLS:** | Y / N |
|  | 8.5 A Hausman test for exogeneity (or equivalent) was performed and reported where the authors cannot reject the null at 95% confidence. | Y / PY / PN / N / NI |
|  | **Controlled pre/post or difference-in-differences:** | Y / N |
|  | 8.6 The authors used a fixed effects or difference-in-differences multivariate regression. | Y / PY / PN / N / NI |
|  | **Interrupted time series** (Bernal, Cummins, & Gasparrini, 2017): |  |
|  | 8.7.1 The period of implementation of the policy or intervention was well defined, or the authors specified a period of graduate roll-out. | Y / PY / PN / N / NI |
|  | 8.7.2 The authors attempt to control for seasonality through methods such as time stratified models, periodic functions (i.e. Fourier terms), and splines or they provided justification for why seasonality was not an issue. | Y / PY / PN / N / NI |
|  | 8.7.3 The data analysed contains more than one time point before and after the period of implementation of policy or intervention. | Y / PY / PN / N / NI |
|  | 8.7.4 Autocorrelation was assessed by analysing a plot of the residuals and the partial autocorrelation function or deviance residuals over time, using a 95% CI. | Y / PY / PN / N / NI |
|  | 8.7.5 If data are normally distributed, a Breusch-Godfrey test was conducted where the authors could not reject the null at 95% confidence. | NA / Y / PY / PN / N / NI |
|  | 8.7.6 If residual autocorrelation was assessed to be present, a model such as Prais regression or ARIMA model was used in order to adjust for autocorrelation. | NA / Y / PY / PN / N / NI |
|  | 8.7.7 If a Poisson distribution was assumed, over-dispersion was controlled for using a scaling adjustment though a Pearson chi-squared test (utilizing the residual degrees of freedom) assessed at 95% confidence. | NA / Y / PY / PN / N / NI |
|  | **Risk-of-bias judgement** | Low / High / Some concerns |
| **Overall risk of bias** | **Risk-of-bias judgement** | Low / High / Some concerns |

### Section C. Conversion Equations

Note: IV here refers to independent variable, rather than instrumental variable. DV refers to the dependent variable. For any two-stage regressions, the second stage regressor is referenced as the IV. $r$ represents the partial correlation.

1. **Continuous Dependent Variables:**

- **OLS or 2SLS Models with Either Continuous or Dichotomous IVs**
  - Equation 1.1:
    - $r=\frac{{SD}_{x}B}{{SD}_{y}}$
    - Data needed:
      - Unstandardized Regression Coefficient ($B$)
      - Standard deviation of DV ${(SD}_{y})$
      - Standard deviation of IV ${(SD}_{x})$
  - Equation 1.2, if the SD of either x or y are reported separately by treatment and control groups:
    - $SD=\sqrt{\frac{{SD}_{t}^{2}\left( n_{t}-1 \right)+{SD}_{c}^{2}\left( n_{c}-1 \right)+\left( \frac{\bar{x}_{t}-\bar{x}_{c}}{2} \right)(n_{t}+n_{c})}{n_{t}+n_{c}-1}}$
    - $r=\frac{{SD}_{x}B}{{SD}_{y}}$
    - Data needed:
      - Unstandardized Regression Coefficient ($B$)
      - Standard deviation of either DV or IV for treatment group ${(SD}_{t})$
      - Standard deviation of either DV or IV for control group ${(SD}_{c})$
      - Mean of treatment group ${(\bar{x}}_{t})$
      - Mean of control group ${(\bar{x}}_{c})$
      - Treatment group sample size ($n_{t}$)
      - Control group sample size ($n_{c}$)
  - Equation 1.3, if only ${SD}_{y}$ is provided:
    - $d=\frac{B}{{SD}_{y}}$, where $d$ refers to Cohen’s d
    - $r=\frac{d}{\sqrt{4+d^{2}}}$
    - Data needed:
      - Unstandardized Regression Coefficient ($B$)
      - Standard deviation of DV ${(SD}_{y})$
  - Equation 1.4, if only *B* and ${se}_{B}$ are provided:
    - Equations:
      1. $t=\frac{B}{{se}_{B}}$, where $t$ refers to the t-statistic
      2. $r=\frac{t}{\sqrt{t^{2}+df}}$
    - Data needed:
      - T-statistic ($t$) or Unstandardized Regression Coefficient and Standard Error ($B, {se}_{B}$)
      - Residual Degrees of Freedom (sample size minus the number of predictors) ($df$)
  - Equation 1.5, t-test
    - $d=\frac{2ttest}{\sqrt{N}}$
    - $r=\frac{d}{\sqrt{4+d^{2}}}$
    - Data needed:
      - Independent t-test results, assuming n1=n2
      - Total sample size N

1. **Dichotomous Dependent Variables**

- **Logit Models**
  - Equation 2.1: Logit models with dichotomous IV and dichotomous DV
    - Equations:
      1. $B=log(OR)$
      2. $d=B(\frac{\sqrt{3}}{\pi})$
      3. $r=\frac{d}{\sqrt{4+d^{2}}}$
    - Data needed:
      - Unstandardized Regression Coefficient or Odds Ratio ($B$ or $OR$)
- **OLS or 2SLS Models with Dichotomous Independent Variables**
  - Equation 2.2.1: OLS or 2SLS models with dichotomous IV and dichotomous DV (if control group success proportion is presented)
    - Equations:
      1. $a=n_{t}(p_{c}+B)$
      2. $b=n_{t}(1-(p_{c}+B))$
      3. $c=n_{c}*p_{c}$
      4. $d=n_{c}(1-p_{c})$
      5. $r=\frac{\left( ad \right)-(bc)}{\sqrt{(a+b)(c+d)(a+c)(b+d)}}$
    - Data needed:
      - Unstandardized Regression Coefficient ($B$)
      - Treatment group sample size ($n_{t}$)
      - Control group sample size ($n_{c}$)
      - Control group success proportion (i.e. mean) of DV ($p_{c}$)
  - Equation 2.2.2: OLS or 2SLS with dichotomous IV and dichotomous DV (if only overall success proportion is presented)
    - Equations:
      1. $a=n_{t}(p+.5B)$
      2. $b=n_{t}(1-(p+.5B))$
      3. $c=n_{c}(p-.5B)$
      4. $d=n_{c}(1-(p-.5B))$
      5. $r=\frac{\left( ad \right)-(bc)}{\sqrt{(a+b)(c+d)(a+c)(b+d)}}$
    - Data needed:
      - Unstandardized Regression Coefficient ($B$)
      - Treatment group sample size ($n_{t}$)
      - Control group sample size ($n_{c}$)
      - Overall success proportion (i.e. mean) of DV ($p$)
- Probit models
  - Equation 2.3
    - Imputed 0 if regression coefficient=0
    - Otherwise, Equation:
    - $d=\frac{B}{{SD}_{x}}$
    - $r=\frac{d}{\sqrt{4+d^{2}}}$
    - Data needed:
      - Unstandardized Regression Coefficient
      - Standard Deviation of IV or Standard Deviation of IV (disaggregated by treatment and control groups)
- Hazard ratios
  - Equation 2.4
    - $d=\ln\left( HR \right)*\sqrt{6}/\pi$
    - $r=\frac{d}{\sqrt{4+d^{2}}}$
  - Data needed:
    - Hazard ratio
- Risk ratios and Prevalence ratios
  - Equation 2.5
    - $OR=\frac{RR}{1-RR}$
    - $B=log(OR)$
    - $d=B(\frac{\sqrt{3}}{\pi})$
    - $r=\frac{d}{\sqrt{4+d^{2}}}$
  - Data needed
    - Risk Ratio (RR) or Prevalence Ratio (PR)
    - Control group success proportion (i.e. mean) of DV ($p_{c}$)

1. **Standard Errors and Confidence Intervals**

- **Standard Errors**
  - If only the standard error of the coefficient is available:
    - Equation 3.1:
      - ${se}_{r}=\frac{r*{se}_{B}}{B}$, where ${se}_{r}$ refers to the standard error of the Partial correlation
    - Data needed
      - Unstandardized Regression Coefficient ($B$)
      - Standard Error of the Unstandardized Regression Coefficient (${se}_{B}$)
  - If only the 95% confidence intervals for the coefficient are available:
    - Equation 3.2:
      1. ${se}_{B}=\frac{{CI}_{upper}-{CI}_{lower}}{1.96}$, where ${se}_{B}$ refers to the standard error of the unstandardized regression coefficient
      2. ${se}_{r}=\frac{r*{se}_{B}}{B}$, where ${se}_{r}$ refers to the standard error of the Partial correlation
    - Data needed
      - Unstandardized Regression Coefficient ($B$)
    - Confidence intervals of the Unstandardized Regression Coefficient (${CI}_{upper}$, ${CI}_{lower}$)
- **Confidence Intervals**
  - The equation below can apply to either regression coefficients as well as partial correlations:
    - Equation 3.3
      1. $CI=B\pm{se}_{B}\cdot1.96$
